# Transferability of biotic interactions: Temporal consistency of arctic plant–rodent relationships is poor

**DOI:** 10.1002/ece3.4399

**Published:** 2018-09-17

**Authors:** Eeva M. Soininen, John‐Andre Henden, Virve T. Ravolainen, Nigel G. Yoccoz, Kari Anne Bråthen, Siw T. Killengreen, Rolf A. Ims

**Affiliations:** ^1^ UiT The Arctic University of Norway Tromsø Norway; ^2^ Norwegian Polar Institute Fram Centre Tromsø Norway

**Keywords:** contextual sensitivity, plant–herbivore interaction, predictive ecology, spatiotemporal variation, tritrophic food web, trophic interaction

## Abstract

Variability in biotic interaction strength is an integral part of food web functioning. However, the consequences of the spatial and temporal variability of biotic interactions are poorly known, in particular for predicting species abundance and distribution. The amplitude of rodent population cycles (i.e., peak‐phase abundances) has been hypothesized to be determined by vegetation properties in tundra ecosystems. We assessed the spatial and temporal predictability of food and shelter plants effects on peak‐phase small rodent abundance during two consecutive rodent population peaks. Rodent abundance was related to both food and shelter biomass during the first peak, and spatial transferability was mostly good. Yet, the temporal transferability of our models to the next population peak was poorer. Plant–rodent interactions are thus temporally variable and likely more complex than simple one‐directional (bottom‐up) relationships or variably overruled by other biotic interactions and abiotic factors. We propose that parametrizing a more complete set of functional links within food webs across abiotic and biotic contexts would improve transferability of biotic interaction models. Such attempts are currently constrained by the lack of data with replicated estimates of key players in food webs. Enhanced collaboration between researchers whose main research interests lay in different parts of the food web could ameliorate this.

## 1 INTRODUCTION

Predictive modelling is increasingly common in ecology, and statistical models created in one context are often used to predict the state of the system in other contexts, such as other geographical areas or climate regimes (Sequeira, Bouchet, Yates, Mengersen, & Caley, 2018; Thuiller et al., 2013). However, the fast development of predictive ecology calls for caution, as it is not always clear whether the current understanding of ecological processes is comprehensive enough to warrant predictions (Mouquet et al., 2015). In particular, biotic interactions are important determinants of biodiversity distribution (Elith & Leathwick, 2009; Wisz et al., 2013) and their strength is related to the functioning and stability of ecosystems (Bartomeus et al., 2016; Gellner & McCann, 2016). Spatial and temporal variability in biotic interactions is an integral part of food web functioning (Gripenberg & Roslin, 2007; Hunter & Price, 1992; Maron, Baer, & Angert, 2014). Yet, predictive biodiversity models rarely account for spatiotemporal variation in biotic interaction strength, as both the knowledge of the relevant variability and the data necessary to quantify it are usually lacking (Thuiller et al., 2013; Wisz et al., 2013).

A key concept of statistical approach to prediction is transferability, defined by Sequeira et al. (2018) as: “The ability of a model developed for a specific site and/or time and/or taxon to predict biodiversity in a different time or place or for a different taxon defines its transferability.” While several aspects of transferability have been studied (see e.g., Huang & Frimpong, 2016; Randin et al., 2006; Wogan, 2016), biotic interactions have rarely been addressed in this context. Increasing our understanding of variability in biotic interactions would therefore improve our ability to forecast the future state of ecological systems.

Herbivore population cycles have been subjected to a large number of studies—because they may elucidate both how biotic interactions can give rise to complex population dynamics and how populations cycles influence ecosystem functioning (Barraquand et al., 2017; Ims & Fuglei, 2005; Krebs, 2011; Myers, 2018). Cycle amplitude is a very important functional property of herbivore population cycles, as it has strong impacts on trophic levels above and below the herbivore (Yang et al., 2010). Cycle amplitude in some herbivores (i.e., boreal and arctic rodents) can for practical purposes be defined as the abundance at the peak phase of the cycle as the abundance of the low phase normally approaches zero at the spatial scale of conventional sampling plots (Steen & Haydon, 2000). In her most recent review on population cycles, Myers (2018) concluded that cycle amplitude shows considerable variation in time and space in all herbivore taxa that exhibit such cycles and that this is “a remaining mystery” despite near a century of research.

From a theoretical point of view, variation in cycle amplitude can have different causes. For example, it can be caused by the same factors that drive the cycles; that is, complex biotic feedback mechanisms that act with time lags—such as predation or plant‐induced defence (Krebs et al., 2014). Alternatively, variable cycle amplitude could result more simply from the direct influence of environmental variation. As a likely case of the latter possibility, Krebs (2013) proposed that temporally or spatially variable productivity of food plants—determining the carrying capacity of the habitat (*K*)—could underlie the large variation in cycle amplitude observed in small rodents. Indeed, the Ecosystem Exploitation Hypothesis, which considers tritrophic systems across a gradient of productivity, predicts that below a certain threshold of productivity food biomass is a powerful predictor of rodent abundance. Above such productivity threshold, rodent abundance should become decoupled from food plant biomass as predators generally suppress rodent peak abundance below a *K* (Aunapuu et al., 2008; Oksanen, Fretwell, Arruda, & Niemelä, 1981). Yet, *K* is a parameter most often set as constant in models constructed to investigate conditions for rodent cycles to result from predator–prey or plant–herbivore interactions (Hanski, Henttonen, Korpimäki, Oksanen, & Turchin, 2001; Henttonen et al., 2017; Turchin & Batzli, 2001). It should also be noted that *K* is defined differently among different models and empirical studies (Chapman & Byron, 2018).

The above theoretical frameworks focus on vegetation as food for rodents, while the role of nontrophic pathways in the regulation of food web structure is increasingly recognized (Gravem & Morgan, 2016; Kéfi et al., 2012; Kimbro, Byers, Grabowski, Hughes, & Piehler, 2014). Small rodents inhabit a “landscape of fear,” where predators are related to several mechanisms modifying rodent abundance, for example, mortality, apparent competition, reduced reproduction, selection for sheltered habitats (Abrams & Cortez, 2015; Dehn, Ydenberg, & Dill, 2017; Dupuch, Morris, & Halliday, 2014). Hence, rodent population densities are often higher in habitats with abundant shelter (Laundre et al., 2014; but see Dupuch, Morris, Ale, Wilson, & Moore, 2014; Dupuch et al., 2014). Shelter plants may therefore, via their indirect effect of modifying predation risk, set the *K* for rodents as suggested by Birney, Grant, and Baird (1976). The importance of shelter in determining *K* can be expected to be pronounced if rodent abundance is regulated by predation. Although previous studies have addressed how variation in food and cover availability relates to the abundance of small rodents (Batzli & Lesieutre, 1995; Dupuch et al., 2014; Hambäck, Schneider, & Oksanen, 1998), they rarely assess spatial and temporal transferability of their statistical models (but see Morris & Dupuch, 2012).

In principle, transferability of predictive models of biotic interactions should increase if several relationships are considered together. Yet, including a range of interactions and contexts can quickly lead to overly complex models, problems in distinguishing the effects of different variables due to multicollinearities, and/or attempts to “model everything” (Mouquet et al., 2015; Wisz et al., 2013). Thus, it appears especially important to focus on the main a priori well‐established functional links (Kissling et al., 2012; Mouquet et al., 2015; Wenger & Olden, 2012). Nevertheless, even if small rodent population cycles have been subjected to many theoretical and empirical analyses, we doubt that the current knowledge of these links is sufficient for developing complex hypotheses on drivers of peak‐phase abundance. We therefore here focus on the simple hypotheses presented by Krebs (2013) and Birney et al. (1976), assessing the spatial and temporal consistency of the proposed relationships between rodent peak density and biomass of food and shelter plants.

We used long‐term monitoring data from an arctic food web where rodents, food plants, and shelter plants have pronounced spatial and temporal variations. We asked (a) whether biomass of food and shelter plants was consistently related to rodent peak abundances within a given population cycle and (b) to what extent the models from the first peak was transferable in time; that is, were we able to predict rodent peak abundance during a population peak based on model created during a previous peak. We thus apply the framework of near‐term forecasting which states that checks of model transferability should be incorporated as a systematic part of long‐term monitoring programmes to make ecology a more predictive science (Dietze, 2017; Dietze et al., 2018).

## 2 METHODS

**Figure 1:**
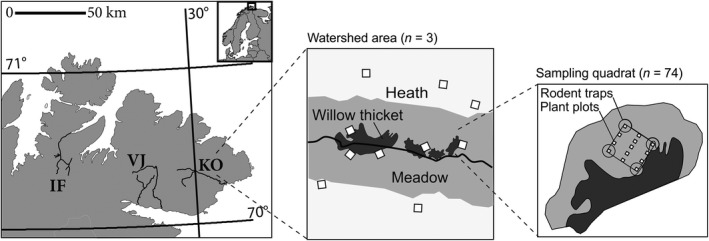
Sampling design for monitoring small rodent and vegetation in three watershed areas (IF = Ifjordfjellet, VJ = Vestre Jakobselv, KO = Komagdalen) in low‐arctic tundra of northeastern Norway. In each watershed area, replicate 15 × 15 m sampling quadrats (white squares; middle inset) are located in heath and meadow habitat. In each sampling quadrat, we annually estimated plant biomass (small squares within large square; right inset) according to Bråthen and Hagberg (2004) and recorded small rodent abundance with traps in the corners according to Myllymäki et al. (1971)

**Figure 2:**
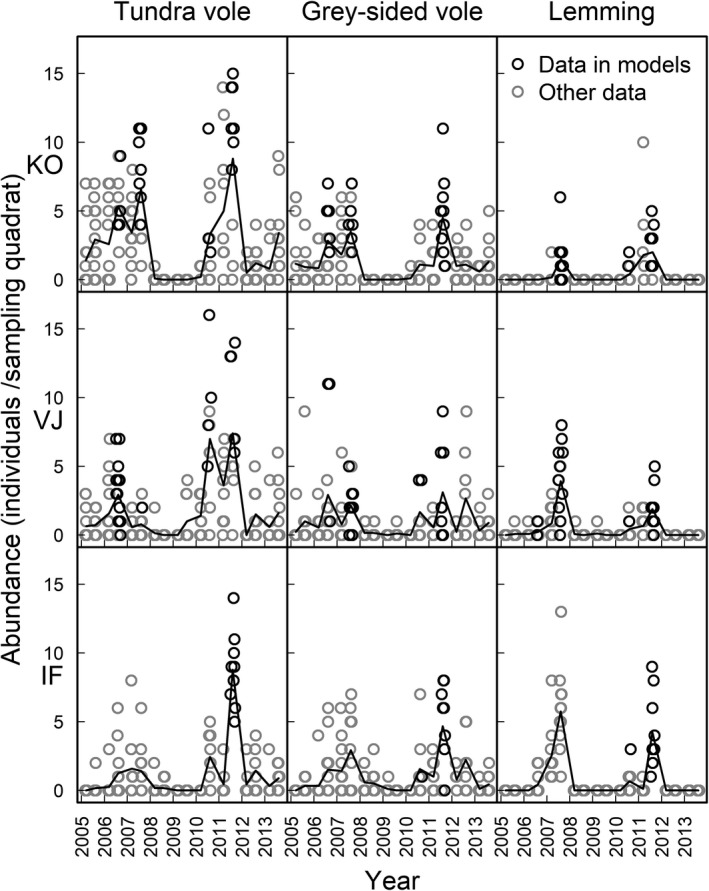
Rodent abundance (no. individuals/sampling quadrat) from 2005 to 2013. Data from primary habitats; meadow for tundra voles, heath for grey‐sided voles and lemmings. Vertical panels show the three different species of the study area, while horizontal panels show three watershed areas. Points are jittered along *x*‐axis to visualize data where several quadrats had same value. Lines go through season‐specific mean abundances. Black circles show data included in the analyses with plant biomass data, while grey circles show data excluded from these analyses (representing data collected during spring, nonpeak years, less‐preferred habitat of the focal species or times when no plant biomass data was available)

This study was conducted in the shrub tundra vegetation zone of northern Norway (70‐71°N to 27‐31°E, Figure 1). The most prominent habitat type of the study area is dwarf‐shrub heath, which is the primary habitat of grey‐sided voles (*Myodes rufocanus*). In addition, the Norwegian lemming (*Lemmus lemmus*) is abundant in the heath habitat during their peak years (Ims, Henden, Thingnes, & Killengreen, 2013). In riparian plains, vegetation is substantially lusher and consists of a mosaic of meadows and willow thickets. These are primary habitats of the tundra vole (*Microtus oeconomus*) (Henden, Ims, Yoccoz, Sørensen, & Killengreen, 2011). Vegetation composition within both habitats exhibits profound spatial variation in the study area and ranges from dominance of palatable plants to dominance of nonpalatable plants (Ravolainen, Bråthen, Ims, Yoccoz, & Soininen, 2013; Soininen, Ravolainen, et al., 2013). Rodent populations in the region normally exhibit a synchronous 4‐ to 5‐year cycle (Figure 2., Terraube et al., 2015). The tritrophic food web of the region is described in detail in Ims, Jepsen, Stien, and Yoccoz (2013), and summary statistics describing various abiotic (e.g., temperature and precipitation) and biotic (e.g., main predators and other herbivores) factors are given in Supporting Information Appendix S1; Table S1.

### 2.1 Study design

We used long‐term monitoring data from the Climate‐Ecological Observatory for Arctic Tundra (COAT) (Ims, Jepsen, et al., 2013). Integrated ecosystem monitoring related to COAT has been conducted since 2005, in three *watershed* areas (Figure 1). Two of these, Komagdalen (KO) and Vestre Jakobselva (VJ), are at the Varanger peninsula at an approximate distance of 40 km from each other, while the third one, Ifjordfjellet (IF), is ca. 100 km further west. Within each of the three watershed areas, data on small rodent abundance and plant biomass is collected in 15 × 15 m *sampling quadrats* (*n* = 18 to 26 per watershed area; Supporting Information Appendix S1 Table S2). The quadrats are small enough to have relatively homogenous vegetation, but large enough to encompass parts of several rodent home ranges (Andreassen, Hertzberg, & Ims, 1998; Ims, 1987) and be representative of demographic processes (Andreassen & Ims, 2001). They are distributed in equal numbers in both heath and meadow habitats with at least 160 m between neighbouring quadrats (Figure 1), in order to the quadrats to be independent, that is, the sampled rodents interact only with the local plants and not those of the of the neighbouring quadrat. Thus, we sampled local rodent abundance across the existing variability of plant biomass in tundra landscapes. In this study, we used data spanning from 2005 to 2013. During this period, small rodent populations peaked twice: 2006–2007 (hereafter *first peak)* and 2010–2011 (hereafter *second peak*) (Figure 2). During the first peak, data on rodent abundance was collected in all watershed areas, while data on plant biomass was collected in KO and VJ only. Effort was reallocated between the two peaks (in 2009) to include plant biomass measurements also at IF and by removing some quadrats from the study design. The number of quadrats included in analyses of plant–rodent interactions was 50 for the first peak and 56 for the second peak (details in Supporting Information Appendix S1; Table S2).

### 2.2 Small rodent abundance

Small rodent populations were sampled twice a year over two trap‐nights (July and September), using the sampling quadrats as small quadrats following the snap‐trapping procedure described by Myllymäki, Paasikalio, Pankakoski, and Kanevo (1971) (Supporting Information Appendix S4, Text S6). We used the number of rodents per species per quadrat as an index for local population abundance, using for each species’ data from the habitat where they were most abundant (Figure 2, Supporting Information Appendix S1; Figure S1). Analyses for grey‐sided voles and lemmings are thus based on data from heath habitat and analyses for tundra voles use data from meadow habitat. We expect that the peak abundance of rodents in the secondary habitats is more related to overspill from primary habitats (Soininen et al., 2014) than to a local interaction with vegetation. Furthermore, our dataset for secondary habitats contains a large number of zeros. Thus, we chose to focus our analyses on the primary habitats.

As plant biomass data was collected after the rodent sampling in July, we only included rodent abundance data from the September trapping in the analyses of plant–rodent interaction. The September sampling also normally represents the annual rodent peak abundance in the shrub tundra zone of Fennoscandia (Ekerholm, Oksanen, & Oksanen, 2001; Ims et al., 2011).

The small rodent population peak in the region lasted for 2 years (Figure 2). Therefore, we used for each quadrat and rodent species data from the year when the quadrat‐specific number of individuals peaked. When the quadrat‐specific rodent abundance was equally high during two subsequent years, we used data from the year when the rodent populations peaked on average in a given watershed area (i.e., during first peak 2007 for KO and 2006 for VJ; during second peak 2011 for all watershed areas).

### 2.3 Plant functional group biomass

Data on plant biomass was sampled annually using the point intercept method according to Bråthen and Hagberg (2004). During *the first peak*, we recorded biomass during the last week of July and the first week of August, that is, at maximum plant biomass. In each quadrat, 13 plots (50 cm × 50 cm) were sampled using 20 points (Figure 1; see Ravolainen et al., 2013). During *the second peak*, we recorded biomass in early September, that is, before substantial withering of plants and concurrently with vole trapping. In each sampling quadrat, 24 plots (50 cm × 50 cm) were sampled using three points. These changes in resolution and timing of the measurements had little effect on the measured biomass (Supporting Information Appendix S2, Text S1, Figure S4). We transformed counts of hits to a quadrat‐specific estimate of biomass g/m^2^ using growth form specific conversion factors described by Ravolainen et al. (2010).

We defined plant functional groups for each rodent species separately (Table 1, Supporting Information Appendix S3). In palatable plant groups, we included plant species that are important food items for the focal rodent species in the study area (Soininen, Ravolainen, et al., 2013; Soininen, Zinger, et al., 2013). In shelter plant groups, we included plant species that have a growth form that has the potential to provide shelter from predators. Plant groups with a similar name have partly different taxonomic composition depending on the rodent species in question (see Table 1). We therefore denote the groups with rodent species‐specific subscripts (e.g., forbs_TV_, forbs_GV_, and forbs_L_ for tundra voles, grey‐sided voles, and lemmings, respectively).

**Table 1:** Plant functional groups used as predictor variable for each rodent species. “Composition” describes which taxa were included in the group and “Function” describes the extent to which food and shelter was likely provided by the plant group

Growth form	Plant functional group	Tundra vole		Grey‐sided vole		Lemming	
		Composition	Function	Composition	Function	Composition	Function
Forbs	Forbs	All forbs—excluding *Rumex acetosa*	Preferred food, shelter (forbs in meadow habitats can be large)	All forbs	Preferred food, unlikely shelter (forbs in heath habitat are small)	All forbs	Food, unlikely shelter (forbs in heath habitat are small)
	*Rumex*	*Rumex acetosa*	Preferred food, unlikely shelter	NAF (included in forbs)		NAF (included in forbs)	
Shrubs	Shelter shrubs	Betulaceae, Ericaceae	Shelter, occur rarely in diets, not selected for	Betulaceae, evergreen Ericaceae, Salicaceae	Shelter, occur in diets but not selected for	Betulaceae, Ericaceae	Shelter, occur rarely in diets
	Palatable shrubs	Salicaceae	Preferred food, shelter	Deciduous Ericaceae	Preferred food, shelter	Salicaceae	Food, shelter
Graminoids	Palatable grasses	Poaceae excluding silica‐rich species	Preferred food, unlikely shelter	NAF	Minor diet component	Poaceae excluding silica‐rich species	Food, unlikely shelter
	Shelter grasses	Tussock‐forming grasses (*Deschampsia cespitosa*)	Shelter	NAF	Low abundance in heath	NAF	Low abundance in heath
	Sedges	NAF		NAF		Cyperaceae, Juncaceae	Food

*Note*

NAF: not analysed for.

### 2.4 Statistical analyses

#### 2.4.1 Temporal consistency of spatial variation within trophic levels

We plotted the quadrat‐specific data from the first peak versus the second peak for both plant biomass and rodent abundance separately. We then tested to what extent the spatial variability observed during the two peaks was temporally consistent using Spearman's rank correlation tests. Furthermore, to assess whether the rodent species exhibited similar dynamics over the two peak summers we calculated (a) Spearman's correlation between the July and September censuses within peaks, (b) the quadrat‐specific summer growth rates (*R=log(N*_*Sept*_ *+1)‐log(N*_*July*_ *+1)*), and (c) the density dependence of the growth rates for each of the two cyclic peaks. Density dependence was estimated based on a state‐space model where measurement error was explicitly incorporated by using the two trapping days for each sampling quadrat as temporal removal occasions (Kéry & Royle, 2016) (Supporting Information Appendix S4, Text S7).

#### 2.4.2 Interaction between trophic levels

The effect of plant biomass on rodent abundance is likely to be a saturating function both on biological grounds (e.g., spacing behaviour and herbivore functional response) and because of the limits of the sampling process (the number of trap‐nights [24] per trapping session sets a maximum). Exploratory plotting supported this expected nonlinearity between rodent abundance and plant biomass. We therefore analysed the abundance of each rodent species as a response variable with generalized linear models (GLM with Poisson distribution; log‐link function) with log‐transformed plant biomass variables as predictors.

Using data from the first peak, we started from models containing the additive effects of all food and shelter variables (Table 1). From the full model, we formed four to five a priori candidate models (Burnham & Anderson, 2002) for each species (Supporting Information Appendix S4; Table S9), and included the null model (i.e., model with only intercept) among the set of models. However, when biomass of two plant groups was correlated (Spearman's rank correlation, ρ > 0.6), they were not included in the same model to prevent potential problems related to collinearity. We used AICc (tundra vole and lemming) and QAICc (grey‐sided vole, due to overdispersion, ĉ ~1.6) (Burnham & Anderson, 2002) to compare candidate models and subsequently identify the “best” models, that is, models with the lowest value of AICc/QAICc (Supporting Information Appendix S4; Table S9). We also estimated model parameters based on model averaging (Burnham & Anderson, 2002), with similar results to the “best” model approach (Supporting Information Appendix S2; Text S2 and Table S3).

#### 2.4.3 Spatial and temporal transferability of trophic relationships

We used the “best” model for each rodent species from the first peak to predict quadrat‐specific vole abundance both *spatially* and *temporally*; that is, assessing the spatial and temporal transferability of the models. We assessed *spatial transferability* using data from the first peak. We sampled randomly, without replacement, half of the sampling quadrats and estimated the model parameters based on the plant biomass data of these quadrats. We then used this model to predict rodent abundance in the remaining quadrats based on plant biomass data. This procedure was repeated 1,000 times for each model. We assessed *temporal transferability* by testing how well the “best” model for the first peak predicted rodent abundance during the second peak. We used the first peaks’ models parametrized with data from all quadrats and predicted the rodent abundance based on plant biomass data sampled during the second peak.

We used the mean absolute error (MAE) (Fielding & Bell, 1997) between predicted and observed values as a simple measure of model transferability. It is interpreted at the scale of observations, that is, in our case rodent abundance measured as number of individuals. We first calculated prediction error based on raw abundances (X) (hereafter MAE_RAW_ *)*:

$${\text{MAE}}_{\mathrm{raw}}\;=\;\frac{\sum_{i=1}^{Q}\left|X_{i,\mathrm{pred}}-X_{i,\mathrm{obs}}\right|}{Q},$$

where Q is the total number of quadrats, *X*_*i*,pred_ is the predicted abundance of rodents in the sampling quadrat *I*,* X*_*i*,obs_ is the observed abundance of rodents in the sampling quadrat *I*.

In order to assess our ability to predict the relative differences in rodent densities between sampling quadrats irrespective of potential differences in the average yearly density (i.e., focusing on spatial variability within a year), we also calculated prediction error based on abundances centred on their mean (hereafter MAE_RELATIVE_):

$${\text{MAE}}_{\mathrm{relative}}\;=\;\frac{\sum_{i=1}^{Q}\left|\left(X_{i,\mathrm{pred}}-\bar{X_{\mathrm{pred}}})-(X_{i,\mathrm{obs}}-\bar{X_{\mathrm{obs}}}\right)\right|}{Q},$$

where $\bar{X_{\mathrm{pred}}}$ and $\bar{X_{\mathrm{obs}}}$ are the average predicted and observed densities observed in the predicted year. The difference between MAE_RAW_ and MAE_RELATIVE_ is thus caused by over‐ or underpredicting the average abundance.

To evaluate the measures of predictive ability obtained for our models, we calculated the above‐described measures of prediction error also for (a) null models and (b) models with minimum obtainable prediction error. Because of the Poisson distributed response variable, the difference between observed and predicted values even for 100% predictive power would never reach zero (Cox & Wermuth, 1992). Therefore, we used simulations to determine the minimum obtainable error, given the range of abundance and sample sizes in the current study (Supporting Information Appendix S4; Text S8).

The dataset that we used for assessment of temporal transferability included all sampling quadrats for which data on plants and rodents were available during the second peak, and hence, also quadrats that were not part of the original dataset used to build the models. However, excluding these quadrats led to no consistent change in transferability (Supporting Information Appendix S2 Table S5) and we therefore chose to retain them in the analyses.

We used the software R (R Development Core Team 2014) for all analyses.

## 3 RESULTS

### 3.1 Spatial and temporal variability within trophic levels

**Figure 3:**
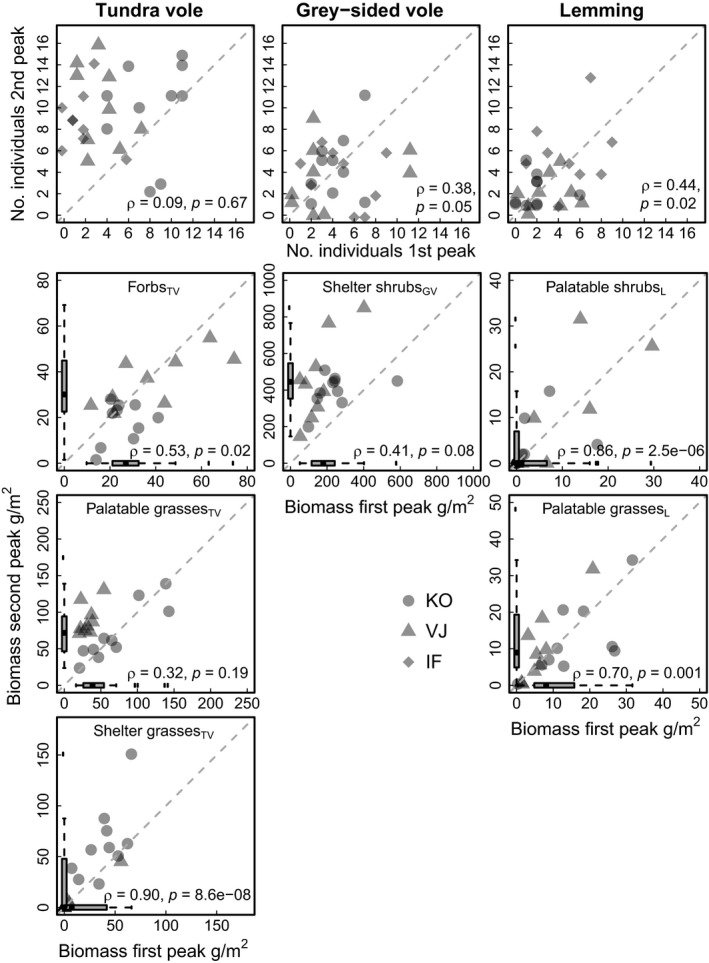
Temporal consistency of spatial variation within trophic levels. Quadrat‐specific rodent abundance (a) and plant biomass (b) in the first rodent peak (x‐axis) are plotted against the second peak (y‐axis). Statistical assessments of the relative temporal consistency of the spatial variation among quadrats are provided by Spearman rank ρ and associated p‐values. Dashed line shows 1 to 1 line; points above this indicate an increase of from first to second peak, while points below indicate a decrease. Symbols denote data from the different watershed areas, note that data from IF is not included in part B of the plot (as no plant data was collected from IF during the first peak). Box plot bars in the plant plots depict the distribution of the biomass estimates for the two rodent peaks, including also data from the quadrats which were sampled only during one peak. Only plant variables selected to enter the statistical prediction model for each rodent species are included in the plots; similar plots for remaining plant variables are given in Supporting Information Appendix *S*4

*Plants:* Biomass of the plant functional groups varied up to 100‐fold among the quadrats during both rodent population peaks and in both habitats (Figure 2, Supporting Information Appendix S1; Figure S3). This spatial variation in plant biomass was temporally consistent for most plant groups; the quadrats with high biomass during the first peak also had high biomass during the second peak (see ρ in Figure 3 and Supporting Information Appendix S1; Figure S3). Yet, biomass of several plant groups increased between the peaks; this was the case for shelter shrubs_GV_, shelter grasses_TV_, and palatable grasses_L_ (Figure 3). In contrast, biomass of forbs_TV_ decreased from the first to the second peak (Figure 3).

*Rodents:* There were no clear differences *between peaks* in the mean, variation, or range of abundances of grey‐sided voles and lemmings, whereas the mean abundance of tundra voles was higher during the second than the first peak (Table 2, Figure 3). Spatial variability in rodent abundances was high during both peaks and somewhat consistent between peaks for grey‐sided voles and lemmings, but not so for tundra voles (Figure 3). Thus, the identity of the quadrats where rodents were abundant differed partly between the peaks. *Within* the population peaks, the abundances of all species increased over the summer in a negatively density‐dependent manner (Table 2, Supporting Information Appendix S1; Figure S2). However, peak summer population dynamics (in terms of growth rate and its negative density dependence) differed somewhat between the peaks. The tundra vole summer growth rate was substantially lower during the first than the second peak, the grey‐sided vole population showed a clear negative density dependence only during the first peak, and the lemmings had weaker density dependence during the first than the second peak (Table 2).

**Table 2:** Characteristics of two small rodent population peaks in northeastern Norway; first peak 2006–2007 and second peak 2010–2011

Data	Statistic	Tundra vole	Grey‐sided vole	Lemming
1st peak	Mean (*SD*) abundance	4.4 (3.3)	3.8 (2.7)	3.5 (3.0)
	Range	0–11	0–11	0–13
	Growth rate (95% CI)	0.44 (0.32, 0.56)	0.91 (0.78, 1.04)	0.72 (0.62, 0.83)
	Density dependence	−0.86 (−0.95, −0.75)	−0.85 (−1.06, −0.48)	−0.59 (−0.88, −0.13)
2nd peak	Mean (*SD*) abundance	9.6 (3.7)	4.3 (3.0)	2.8 (2.2)
	Range	2–16	0–11	0–9
	Growth rate (95% CI)	1.41 (1.26, 1.56)	1.04 (0.92, 1.16)	0.88 (0.71, 1.04)
	Density dependence	−0.97 (−1.00, −0.92)	−0.53 (−0.94, 0.28)	−1.08 (−1.36, −0.88)

Notes

All statistics are based on spatial variation in numbers of individuals trapped per quadrat. Growth rate refers to seasonal growth rate within each peak; that is, quadrat‐specific growth from spring to autumn. Density dependence is calculated as the mean of the posterior of (β_DD_ – 1) from the Bayesian state‐space model (see Supporting Information Appendix S4). 95% credible interval is given in the parentheses.

### 3.2 Relationships between trophic levels

**Figure 4:**
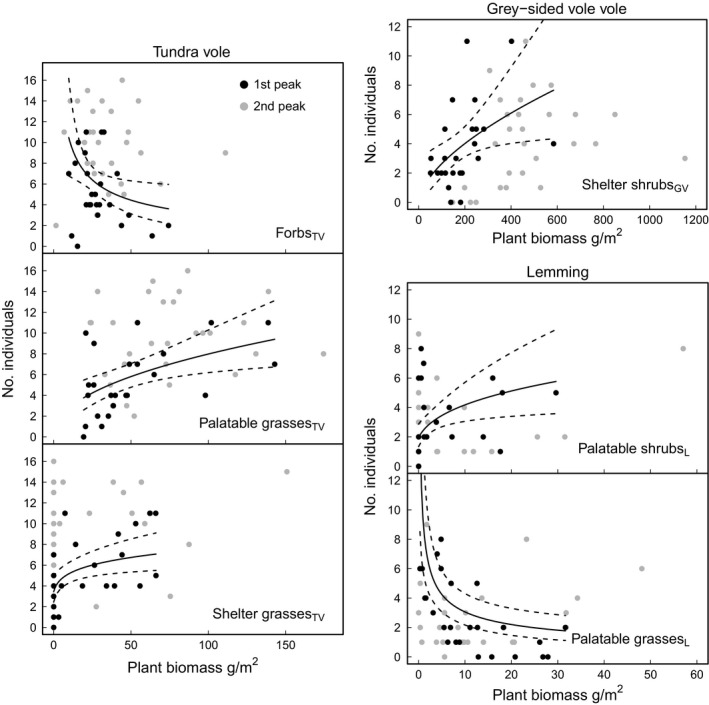
Relationship between plant biomass and rodent abundance. Points show raw data for both peaks (peak 2006–2007 as black points and peak 2010–2011 as grey points) and lines denote predicted values (black lines; predictions, stippled lines; 95% confidence interval of the prediction) for “best” model from the first peak. For each plot, other variables included in the model were centred on their respective means

The “best” model for peak abundance of tundra voles included a negative effect of the biomass of forbs_TV_ and positive effects of the biomass of palatable grasses_TV_ and shelter grasses_TV_ (Table 3, Figure 4). For grey‐sided voles, the “best” model only included a positive effect of the biomass of shelter shrubs_GV_ (Table 4, Figure 4). For lemmings, the “best” model included a negative effect of the biomass of palatable grasses_L_ and a positive effect of the biomass of palatable shrubs_L_ (Table 3, Figure 4).

**Table 3:** Coefficients from the “best” models for tundra vole, grey‐sided vole and lemming abundance. Estimates are on log‐scale. Residual column shows residual degrees of freedom and residual deviance, respectively

Species		Estimate	95% CI	Residual
Tundra vole	Intercept	1.21	−0.30, 2.69	21, 25.51
	Forbs	−0.52	−0.95, −0.10	
	Palatable grasses	0.46	0.16, 0.76	
	Shelter grasses	0.17	0.06, 0.28	
Grey‐sided vole	Intercept	−1.80	−4.36, 0.67	23, 39.55
	Shelter shrubs	0.60	0.13, 1.08	
Lemming	Intercept	1.42	1.01, 1.78	22, 30.57
	Palatable grasses	−0.47	−0.67, −0.28	
	Palatable shrubs	0.32	0.11, 0.52	

### 3.3 Spatial and temporal transferability of trophic relationships

The spatial predictions of our models differed from the observed numbers of individuals with 2.01 to 2.37, representing 18% to 22% prediction error compared to the observed range of the individuals (Table 4, MAE_RAW_). Hence, the models’ prediction errors were reasonably close to the simulated minimum obtainable error, which ranged between 12% and 14% (Table 4).

**Table 4:** Spatial and temporal transferability of predictions for tundra vole, grey‐sided vole and lemming abundance, measured as mean absolute predictive error (MAE ± standard deviation)

Species	Error	Spatial transferability			Temporal transferability		
		Best model	Null model	Simulated	Best model	Null model	Simulated
Tundra vole	Raw	2.37 ± 0.32 (0.20)	2.84 ± 0.14 (0.24)	1.41 ± 0.35 (0.12)	5.26 ± 5.52 (0.31)	4.77 ± 3.08 (0.28)	1.48 ± 0.25 (0.09)
	Relative	2.22 ± 0.31 (0.19)	2.62 ± 0.16 (0.22)		4.19 ± 5.72 (0.25)	3.03 ± 2.06 (0.18)	
Grey‐sided vole	Raw	2.18 ± 0.16 (0.18)	2.23 ± 0.13 (0.19)	1.40 ± 0.36 (0.12)	3.13 ± 2.02 (0.26)	2.47 ± 1.65 (0.21)	1.49 ± 0.24 (0.12)
	Relative	2.02 ± 0.16 (0.17)	2.06 ± 0.18 (0.17)		2.38 ± 1.70 (0.20)	2.45 ± 1.61 (0.20)	
Lemming	Raw	2.01 ± 3.23 (0.22)	2.17 ± 0.26 (0.24)	1.26 ± 0.32 (0.14)	2.16 ± 1.58 (0.22)	1.72 ± 1.39 (0.17)	1.35 ± 0.22 (0.14)
	Relative	1.99 ± 4.34 (0.22)	2.03 ± 0.30 (0.22)		2.18 ± 1.53 (0.22)	1.72 ± 1.39 (0.17)	

Notes

Unit is number of individuals per sampling quadrat. Values in parenthesis show the proportion of the observed range of individuals that the MAE represents. Column “Error” refers to type of predictive error (mean absolute prediction error, see methods for exact definitions). “Raw” refers to MAE_RAW_ (i.e., using raw abundances) and “Relative” to MAE_RELATIVE_ (i.e., error for relative differences in rodent numbers between sampling quadrats irrespective of potential differences in the average yearly density). Column “Best model” refers to mean ± standard deviation of predictive error based on “best” model for each species (cf. Table 3), column “Null model” refers to mean ± standard deviation of predictive error based on null models, and column “Simulated” presents the simulated minimum obtainable prediction error based on perfect fit. Note that the simulation results do not depend on whether data are centred or not and thus error is given only once.

The spatial transferability of the two vole species was better than the temporal transferability (Table 4). The difference between observed and predicted numbers of individuals was 5.26 and 3.13 for tundra voles and grey‐sided voles, respectively (Table 4, MAE_RAW_). This represents 31% and 26% error compared to the observed range of the individuals. However, spatial and temporal prediction errors were similar for the lemmings (Table 4). Yet, temporal predictions based on a null model were consistently better than predictions based on the modelled relationship with plant biomass (Table 4). Hence, even if the lemming model had better transferability than that of voles, it was not able to predict lemming abundance better than a null model that assumes the distribution of lemmings to be independent of the plant variables. Indeed, when model selection was repeated for the data of the second peak only, the plant biomass was little related to rodent abundance, as the null model was ranked among the “best” models for all rodent species (Supporting Information Appendix S2; Text S3 and Table S4). Three lines of evidence thus point towards low temporal predictability of plant–rodent relationships: (a) large predictive errors of our plant‐based models for the two vole species, (b) null models’ ability to predict better than the plant‐based models’ ability, and (c) the fact that plant‐based models were not selected over the null models during the second peak).

**Figure 5:**
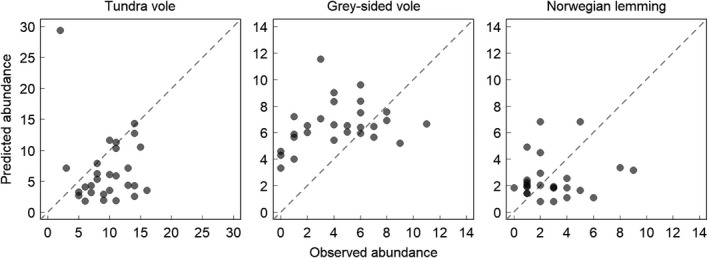
Observed and predicted abundances of rodents during the second peak (2010–2011). Observed quadrat‐specific rodent abundances during the second small rodent population peak compared with corresponding densities predicted by statistical models built on first peaks data

We found little differences in model transferability between raw and relative prediction errors (Table 4). Yet, the model for tundra voles appears to have underpredicted the average vole densities, whereas the model for grey‐sided voles showed an opposite pattern (i.e., over prediction; Figure 5). Hence, the uncertainty of our predictions for these models was partly related to our ability to predict the average abundance levels of voles in the landscape.

## 4 DISCUSSION

We found that the spatial variation in small rodent population peak abundance was consistently related to the availability of food and shelter plants during the first population peak, suggesting that both types of plant functional groups can play a role in shaping rodent abundance. However, some of the relationships between rodent abundance and food plant biomass had a negative sign, that is, rodent abundance decreased as plant biomass increased. Such relationships are unlikely to result from simple one‐directional bottom‐up effects of plant biomass on herbivore peak abundance. Moreover, temporal transferability of the models to the second peak was poorer, suggesting that plant biomass has a complex relationship with tundra rodent abundances. These results demonstrate that there can be considerable heterogeneity in the strength of food web relationships even in relatively simple systems.

There may be various reasons for the lack of temporal transferability that we found. Importantly, the role of vegetation in the dynamics of tundra rodents is likely a more complex interaction than a one‐directional effect of food or shelter. Indeed, strong negative impact of rodents on plant biomass has been previously demonstrated for the shrub tundra zone (Olofsson, te Beest, & Ericson, 2013; Ravolainen, Bråthen, Yoccoz, Nguyen, & Ims, 2014) and is indicated by the negative relationship we found between palatable plants and the abundance of tundra voles and lemmings. While a negative effect of food could also result from rodents trading off food for shelter, this seems unlikely as food plant biomass had no strong negative correlations with shelter plant biomass. Our findings on tundra voles’ growth rate and their food plants also exemplify the complexity of plant–rodent relationships. During the second peak, tundra voles’ growth rate was stronger and the forb biomass lower than during the first peak. Such a pattern creates an expectation of a stronger negative relationship between tundra voles and forbs during the second than first peak—exactly the opposite to our findings.

Temporal inconsistency in a plant–herbivore interaction may result from interannual variability in the abiotic or biotic contexts determining plant growth (e.g., temperature and precipitation; cf. Gauthier et al. (2013), van der Wal and Stien (2014) or reindeer grazing; c.f. Ravolainen et al. (2011))—an example of contextual sensitivity (cf. Van Bavel, Mende‐Siedlecki, Brady, & Reneiro, 2016). For instance, rodent grazing may be able to control biomass of food plants in years of poor growing conditions or heavy reindeer grazing (i.e., causing a negative relationship between rodent abundance and plant biomass). In contrast, plants may compensate for rodent grazing under benign growing conditions or light reindeer grazing (i.e., causing either no or even positive relationships). Such dynamic interaction would likely be better captured by measurements of plant productivity than snapshot data of plant biomass, in turn probably improving transferability of models describing plant–herbivore interactions.

Another likely reason for the lack of temporal transferability that we observed is that tritrophic interactions can create more complex relationships between plants and herbivores (Holt & Barfield, 2013). Potentially strong top‐down impacts of predators were not included in our models, and it is very likely that also this have contributed to the lack of temporal model transferability. Within the substantial body of literature focusing on predator–prey interactions of tundra rodents (e.g., Gilg, Hanski, & Sittler, 2003; Hanski et al., 2001; Therrien, Gauthier, Korpimäki, & Bêty, 2014), some models predict that prey carrying capacity or prey refuges may play a role in shaping rodent cycle amplitude (Hanski & Korpimäki, 1995; Turchin & Hanski, 1997). Prey carrying capacity can be interpreted as food plants and prey refuges as shelter plants, but these models are not tailored to address differences between spatial and temporal settings and provide therefore little aid for interpreting the variability we observed. Nonetheless, the sheltering effect of vegetation may depend on variable abundances of mobile avian predators (Pokrovsky et al., 2014). Spatiotemporal variation in predation risk by avian predators could therefore introduce corresponding variation in the relationship between shelter plants and rodents.

Herbivore biomass in the productive tall shrub tundra zone is predicted to be uncoupled from plant biomass due to strong top‐down regulation by predators (according to EEH; Oksanen et al., 1981; Aunapuu et al., 2008). Our results from the first peak show that plants and rodents can, however, have a clear relationship. This apparent discrepancy can be explained by that while the EEH considers conditions at equilibrium, predator–prey interactions are in reality liable to impacts of environmental stochasticity (Hanski & Korpimäki, 1995). Such stochasticity can give scope for clearly manifested plant–rodent interactions also in productive tundra habitats, at least when the regulating force of predation is small, as also indicated by Hoset et al. (2017). The spatial setting of rodent–predator interactions for a given species of rodent may be by further complicated if the presence of another rodent species attracts predators (Henttonen, Oksanen, Jortikka, & Haukisalmi, 1987; Ims et al., 2013) or if the role of vegetation for rodents is not limited to food, as exemplified by our study. Hence, expectations of EEH may well apply during one cyclic peak but may be overruled by other factors (not considered in the EEH framework) in other peaks.

However, modelling several trophic interactions and their interdependencies within the same food web simultaneously is challenging. In particular, appropriate datasets are mostly lacking as few datasets cover several food web components at a fine resolution, over long timescales, and across replicated locations. For instance, in spite of using data from an extensive food web research programme (Ims et al., 2013), we currently lack adequate data on predators and plant productivity (as opposed to plant biomass) for incorporating them into predictive models. In addition, when different interactions occur at different spatial scales, addressing them with common models requires careful work. In addition, different interactions are likely to occur at different spatial scales. For example, rodent–plant interactions occur at the scale of the home range of a rodent (a few tens of metres) whereas predators interact with rodents at a larger scale (hundreds of metres to kilometres), many rodents home ranges being affected by the same predator. Yet, neither the theory of food web ecology nor empirical modelling tools address such variability of scales adequately.

Our study also exemplifies the challenge of making temporal predictions based on spatial data. The range of several plant biomass variables differed between the peaks (Figure 3), as could be expected based on the few time series that describe annual variation in Arctic plant biomass (Gauthier et al., 2013; Olofsson et al., 2013; van der Wal & Stien, 2014). Thus, longer temporal extent may be required to capture the whole span of the variability of plant biomass existing through time. This fits well with the fact that our predictions for vole abundances had systematic errors (Figure 5). The temporal variation in plant biomass may also partly explain why we found consistently better spatial than temporal transferability. We tested the spatial transferability based on a dataset drawn from the original range of plant biomass data, the range of plant biomass thus remaining similar to the one used for developing the model. Moreover, the temporal transferability did not depend on the spatial extent of the dataset during the second peak (Supporting Information Appendix S2, Table S5). The spatial extent of our data appears therefore sufficient to encompass the relevant range of plant biomass in the region.

Predictor variable range change is a fundamental problem for transferability in ecology (Randin et al., 2006; Thuiller, Brotons, Araujo, & Lavorel, 2004). In particular, as ecological relationships are rarely linear, sampling different ranges of predictor variables leads easily to different models. Indeed, given that most ecological systems are currently subjected to rapidly changing climatic conditions (possibly leading to “novel climates”), numerous environmental and biological variables are expected to change their ranges to unknown extents. At a decadal temporal extent, both the range of several variables and the relationship between them can change, as illustrated by Morris and Dupuch (2012) for lemming habitat selection. Hence, it is debatable to what extent numeric models are able to forecast the future states of ecological systems that are also more liable to transient dynamics (Petchey et al., 2015; Planque, 2016). Nevertheless, making model validations and predictions—even based on relatively short‐term data as performed in the present study—is a valuable approach for effective learning about the functioning of ecological systems (Dietze, 2017; Dietze et al., 2018).

## 5 CONCLUSION

The pronounced spatiotemporal variation in the relationship between rodent and plants implies that snapshots of spatial variability may not be able to provide generalizable conclusions about biotic interactions. Indeed, we would have drawn rather different conclusions about the role of plant functional groups for rodents if using data from the first or the second peak only. We propose that predictions of biotic interaction strength may be improved by parameterizing a more complete set of a priori determined key functional links within the food web and the impacts of the abiotic environment. A crucial limitation for achieving such models is the scarcity of fine‐grained large‐scale (cf. Wisz et al., 2013) datasets that cover spatiotemporal variation of such key players. This is unsurprising, as such datasets are extremely laborious to achieve. As a potential solution, we suggest strengthened collaboration between researchers whose main research interests lay in different parts of the food web. In particular, employing common study designs that allow for combining datasets across the food web will enable simultaneous modelling of several food web interactions.

## CONFLICT OF INTEREST

None declared.

## AUTHOR CONTRIBUTIONS

All authors contributed to the design of the study. EMS, JAH, VTR, KAB, and STK collected field data. EMS and JAH analysed data. EMS wrote the paper with contributions from all authors.

## DATA ACCESSIBILITY

Data are available through Dryad (doi: 10.5061/dryad.7r5d56c).
