# Supplementary material for: Transferability of biotic interactions: Temporal consistency of arctic plant–rodent relationships is poor
Source: Ecol Evol. 2018 Sep 17;8(19):9697–711. doi: 10.1002/ece3.4399 (PMC6202721; doi:10.1002/ece3.4399)
Supplement: Supplementary file 1 [file ECE3-8-9697-s001.docx]

Supplementary material for: Soininen, E.M., Henden, J.-A., Ravolainen, V., Yoccoz, N.G., Bråthen, K.-A., Killengreen, S. and Ims, R.: Transferability of biotic interactions: temporal consistency of arctic plant-rodent relationships is poor.

**Appendix S1: Supplementary figures and tables on raw data and study design**

**Supplementary Figure S1. Rodent abundance (no. individuals / sampling quadrat) from 2005 to 2013.** Data from secondary habitats; heath for tundra vole, meadow for grey-sided vole and lemming. Vertical panels show the three different species of the study area, while horizontal panels show three watershed areas. Points are jittered along x-axis to visualize data where several quadrats had same value. Lines go through season-specific mean abundances.


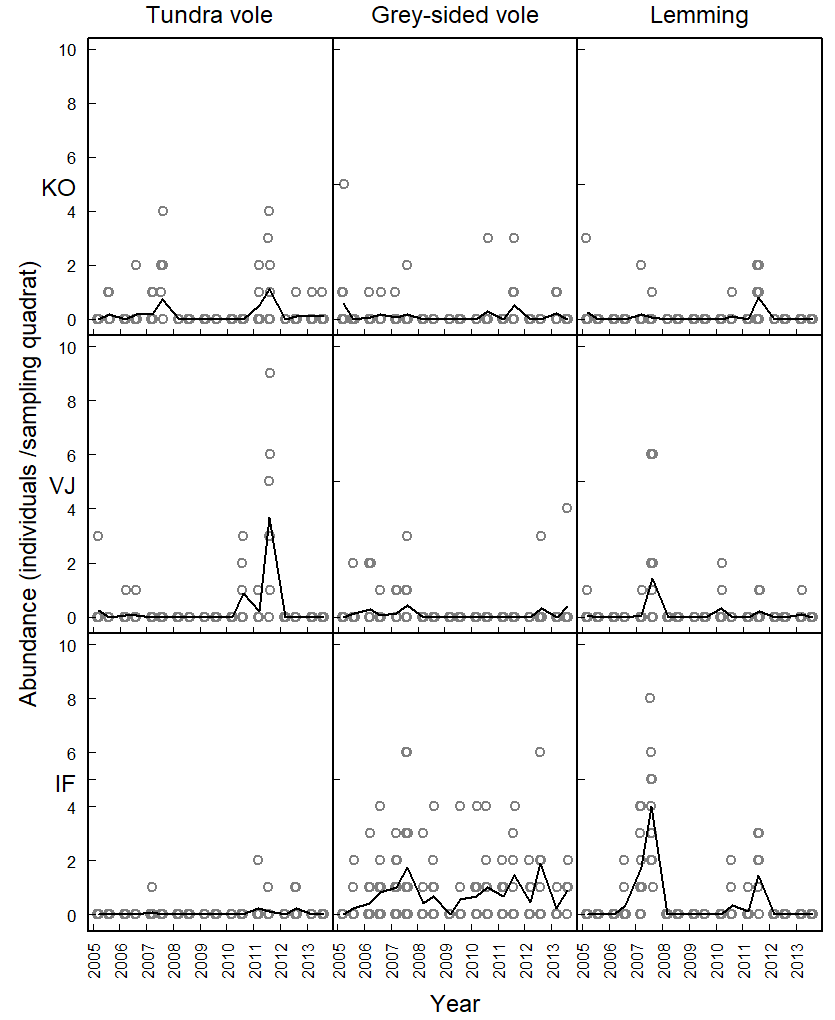


**Supplementary Figure S2.** Temporal consistency in spatial variation of rodent abundance from summer to autumn within the population peaks. At x-axis are quadrat-specific rodent abundances during summer, at y-axis corresponding data during autumn. Dotted line shows 1 to 1 line; points above this indicate an increase of biomass from summer to autumn, points below a decrease. Spearman rank correlation of rodent abundance between summer and autumn (ρ and related p-value) are shown.


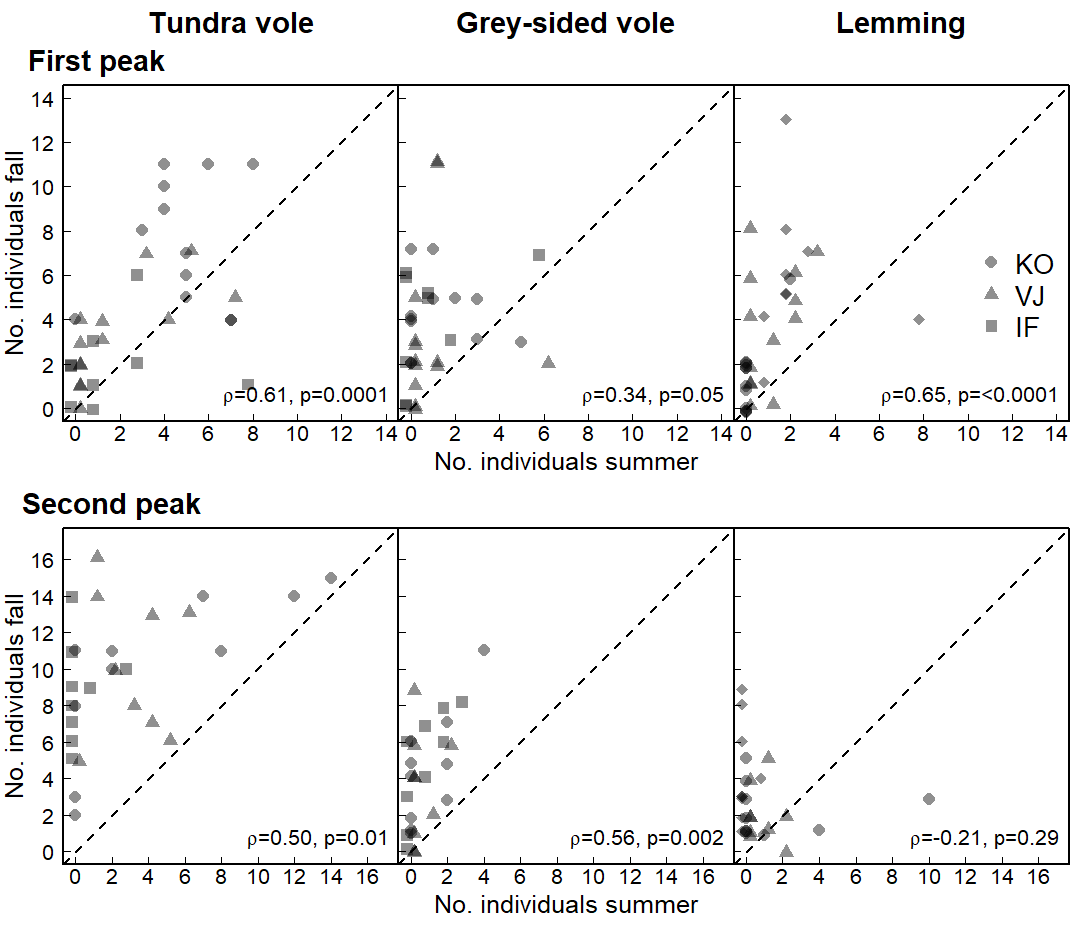


**Supplementary Figure S3.** Temporal consistency in spatial variation of plant functional group biomass; plant groups that were not included in the best models for plant-rodent interactions. Data are arranged in columns according to rodent species; for example below the title “Tundra vole” are plant functional groups used for the analyses for this species abundance. At x-axis are quadrat-specific biomasses (g/m^2^) during the first peak, at y-axis biomasses of the corresponding quadrat during the second peak. Dashed line shows 1 to 1 line; points above this indicate an increase of biomass from first to second peak, points below a decrease. Box plot bars depict the distribution of the biomass estimates for the two rodent peaks, including also data from the quadrats that were sampled only during one of the peaks and could therefore not be included in the correlation. Spearman rank correlation of plant biomass between first and second peak (ρ and related p-value) are shown.


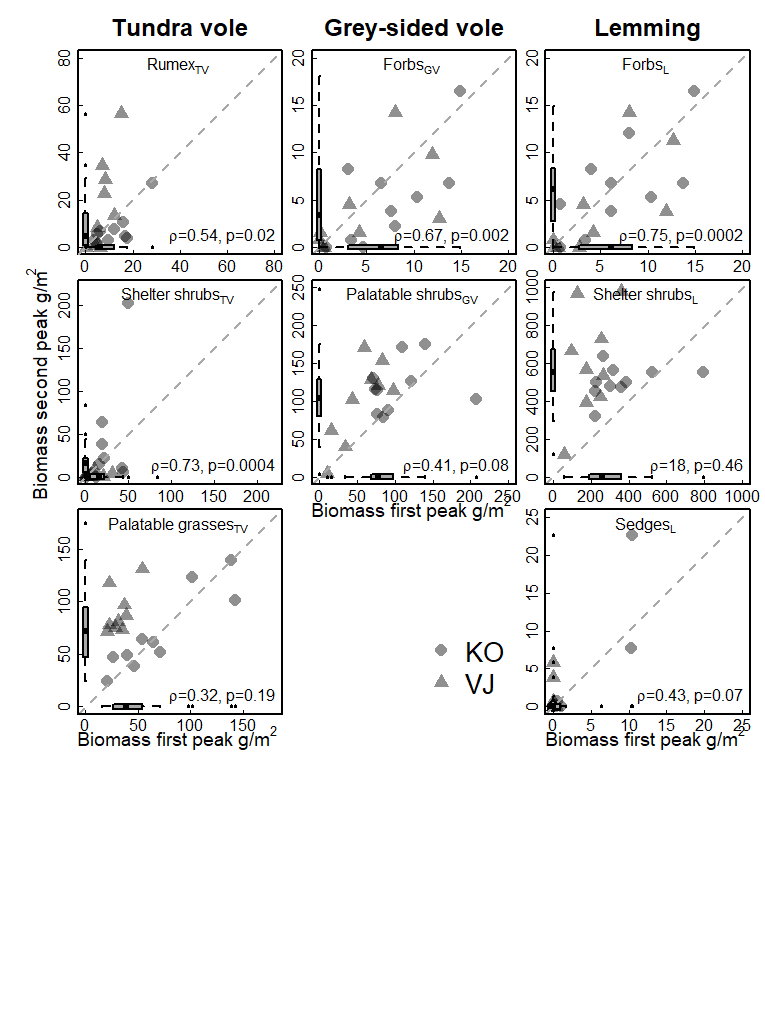


**Supplementary Table S1. Abiotic and biotic context factors in the three watersheds included in this study. * =** Data from Ims et al. 2013, page 113, for IF averaged across the localities used in this study (Upper Stuorrajohka and Estorjohka). ** = Ims et al., unpublished data. $ Data averaged across all sampling quadrats included in this study.

|  | **Komagdalen** | **Vestre Jakobselv** | **Ifjordfjellet** |
| --- | --- | --- | --- |
| **Abiotic contexts** |  |  |  |
| Annual mean temperature °C * | 0.01 | -1.16 | -1.71 |
| Mean annual precipitation sum (mm) * | 636 | 595 | 502 |
| Bedrock * | Rich; slate and limestone dominated types | A mixture of rich (slate and limestone dominated types) and poor (mainly sandstone) types | Rich; slate and limestone dominated types |
| Altitude (m.a.s.l.) $ | 142 | 266 | 286 |
| **Biotic contexts** |  |  |  |
| Small rodent predators ** | Stoat, least weasel, rough-legged buzzard, long-tailed skua, arctic fox, red fox, snowy owl | Stoat, least weasel, rough-legged buzzard, long-tailed skua, arctic fox, red fox, snowy owl | Stoat, least weasel, rough-legged buzzard, long-tailed skua, red fox |
| Reindeer abundance during growing season * | Summer pasture area; animals/km^2^ 1980-2003 = 1.7, 2007-2010 1.8 | Summer pasture area; animals/km^2^ 1980-2003 = 2.5, 2007-2010 = 3 | Mainly migration pasture area; reindeer presence low during summer |
| Other vertebrate herbivores present at low densities | Moose, hare, willow ptarmigan, rock ptarmigan | Moose, hare, willow ptarmigan, rock ptarmigan | Moose, hare, willow ptarmigan, rock ptarmigan |
| Approximate tree limit (m.a.s.l.) * | 25-50 | 200 | 200 |

**Supplementary Table S2.** Number of sampling quadrats included in this study; per watershed, small rodent population peak and habitat. The quadrats at IF were not included in the plant-rodent models during the first peak as no plant biomass data was collected in this watershed prior to 2009. They are, however, included in analyses of rodent abundances only (i.e. within-peak analyses and statistics, Table 2). Effort was reallocated between the two peaks (in 2009) to include plant biomass measurements also at IF and by removing some quadrats the study design. All of the quadrates that were sampled during the second peak were also included in the earlier dataset.

|  |  | **KO** | **VJ** | **IF** |
| --- | --- | --- | --- | --- |
| **1^st^ peak** | **Meadow** | 12 | 13 | *12* |
|  | **Heath** | 12 | 13 | *12* |
| **2^nd^ peak** | **Meadow** | 10 | 9 | 9 |
|  | **Heath** | 10 | 9 | 9 |

**Appendix S2: Additional data analyses**

**Supplementary text S1: Comparison between estimates of plant abundance from two point intercept measurement methods applied during the study**

We changed the point intercept method between the two small rodent population peaks in order to have a more efficient methodology for sampling plant abundance data in the field. However, we conducted a calibration study between the old and new methodology to estimate to what extent this change caused bias to the datasets representing the first and the last small rodent population peak. The change in method caused three levels of change, for which the calibration study addressed the first two:

*First,* we increased the number of plots per quadrate, and reduced the sampling effort per plot. During the first rodent population peak, we recorded data from a total of 260 pins, divided between 13 permanent plots of 20 pins each, whereas during the second peak we used a total of 72 pins divided between 24 plots of 3 pins each. Although we reduced the number of pins by almost 75%, we had a more even distribution of pins across the quadrat. This change in effort had minor effect on the quadrat-specific point intercept frequency measured, with majorly overlapping frequency estimates (Figure S4a and S4b).

*Second,* the measurement period changed from late July to early August to late August. This shift from mid to late season also had minor effect on the point intercept frequency measured, with majorly overlapping frequency estimates (Figure S4c and S4d). Plant biomass in the tundra reaches its peak by late July and does not substantially decrease after that. Hence, we can expect that a biomass measurement during the annual peak biomass and after that reflect similar biomass.

*Third,* the resolution of registrations changed; while plants were recorded using taxonomic groups during the first peak (mainly species, in some cases genus), they were recorded using plant functional groups during the second peak. As these groups were not specifically defined with small rodents in mind, some of the groups include species that are not optimally placed. For example, *Harimanella hypnoides* is recorded together with other evergreen woody dwarf shrubs. Hence, it is grouped together with other species of this group as a “shelter shrub” for the analyses of grey-sided voles even though it is so small that it unlikely provides much shelter. Other species with similar issues are *Arctostaphylos alpina* and *Loiseleuria* *procumbens* which were grouped into shelter shrubs for lemmings (Table S8). However, their combined biomass is very small compared to the combined biomass of other species in these groups (Table S8) and they are thus unlikely to affect our results.

Finally, we assume that the regressions between biomass and point intercept frequency (i.e. number of hits on pins) are representative for both datasets. During the calibration work, we took care to include plots with high and low biomass of different plants. Hence, the plots also covered a range of plant sizes and thus the regressions are based on a good coverage of existing plant sizes and shapes. While the calibrations were done in August (during plant peak biomass), the plant morphometry in September does not differ greatly from that in August. Indeed, the abundance of very young plants (i.e. plants that are not present in August) should not increase towards September.

**Figure S4.** Box-and-whisker plot (with median, quartiles, minimum and maximum values and outliers) of point intercepts measured for the functional groups applied in the study. Comparisons of a) 3-pin vs 25-pin methodology in tundra vole (TV) habitats, b) 3-pin vs 25-pin methodology in grey-sided vole (GV) and lemming (LE) habitats, c) mid and late season measurements in tundra vole (TV) habitats, and d) mid and late season measurements in grey-sided vole (GV) and lemming (LE) habitats.

**Figure S4a**

**
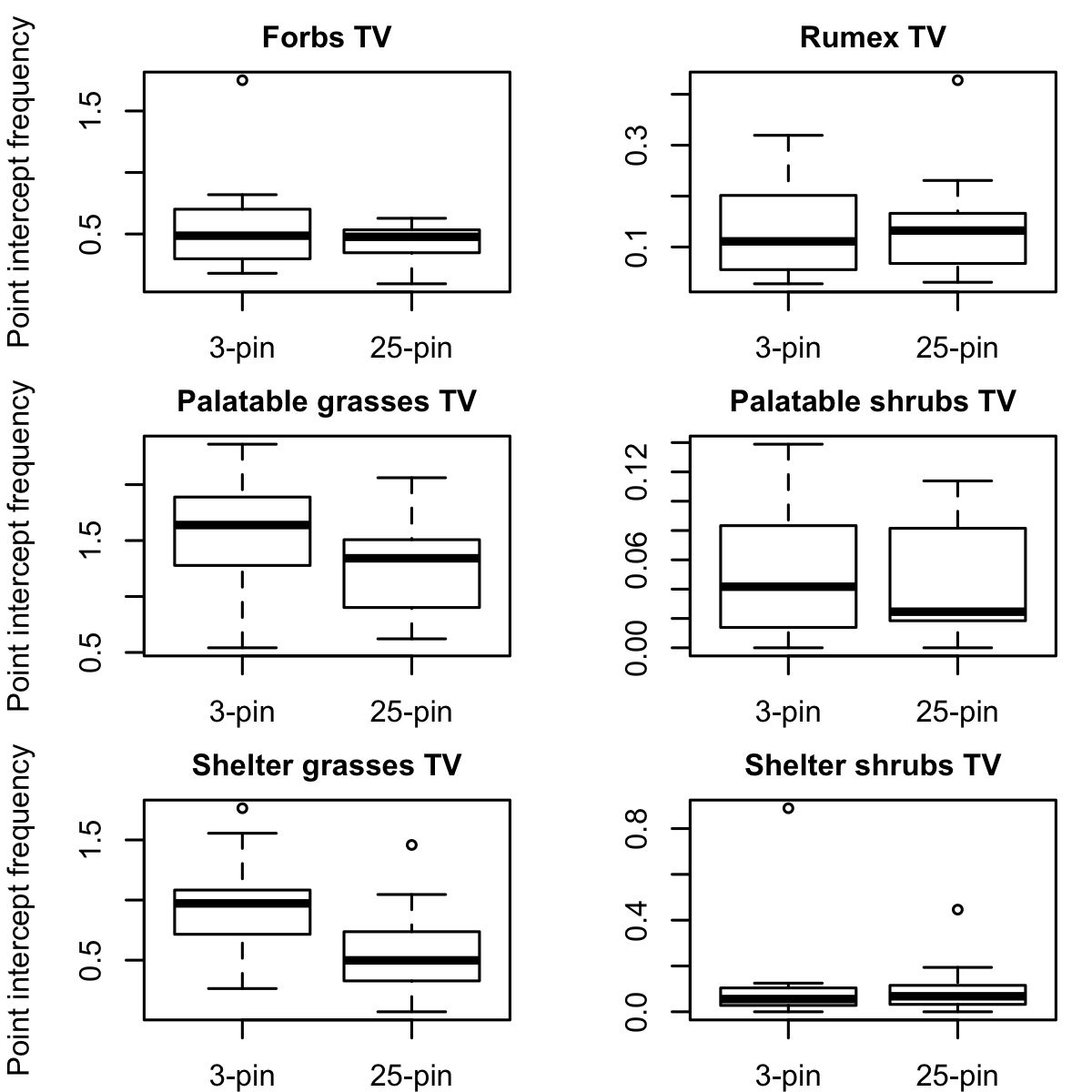
**

**Figure S4b**

**
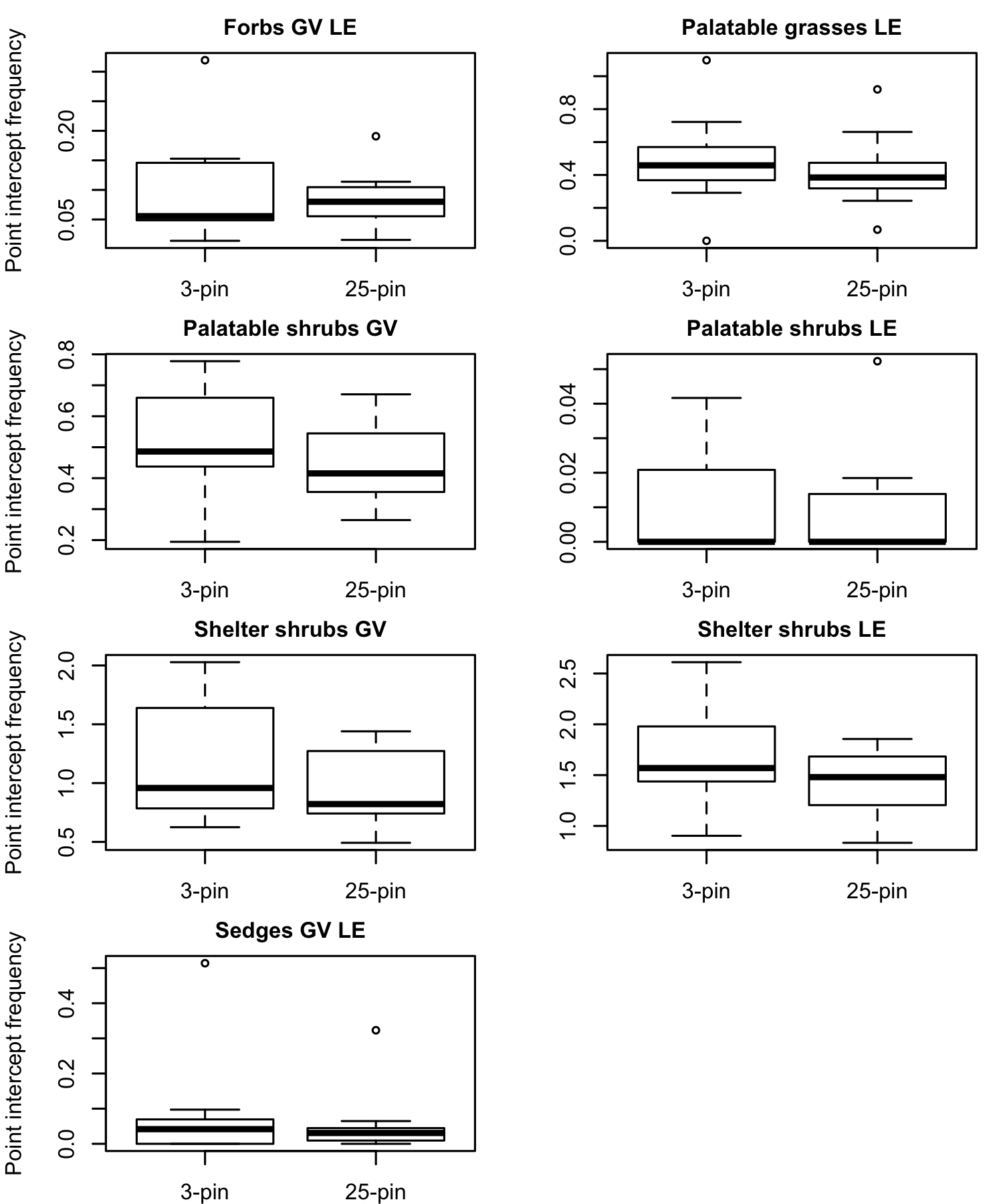
**

**Figure S4c**

**
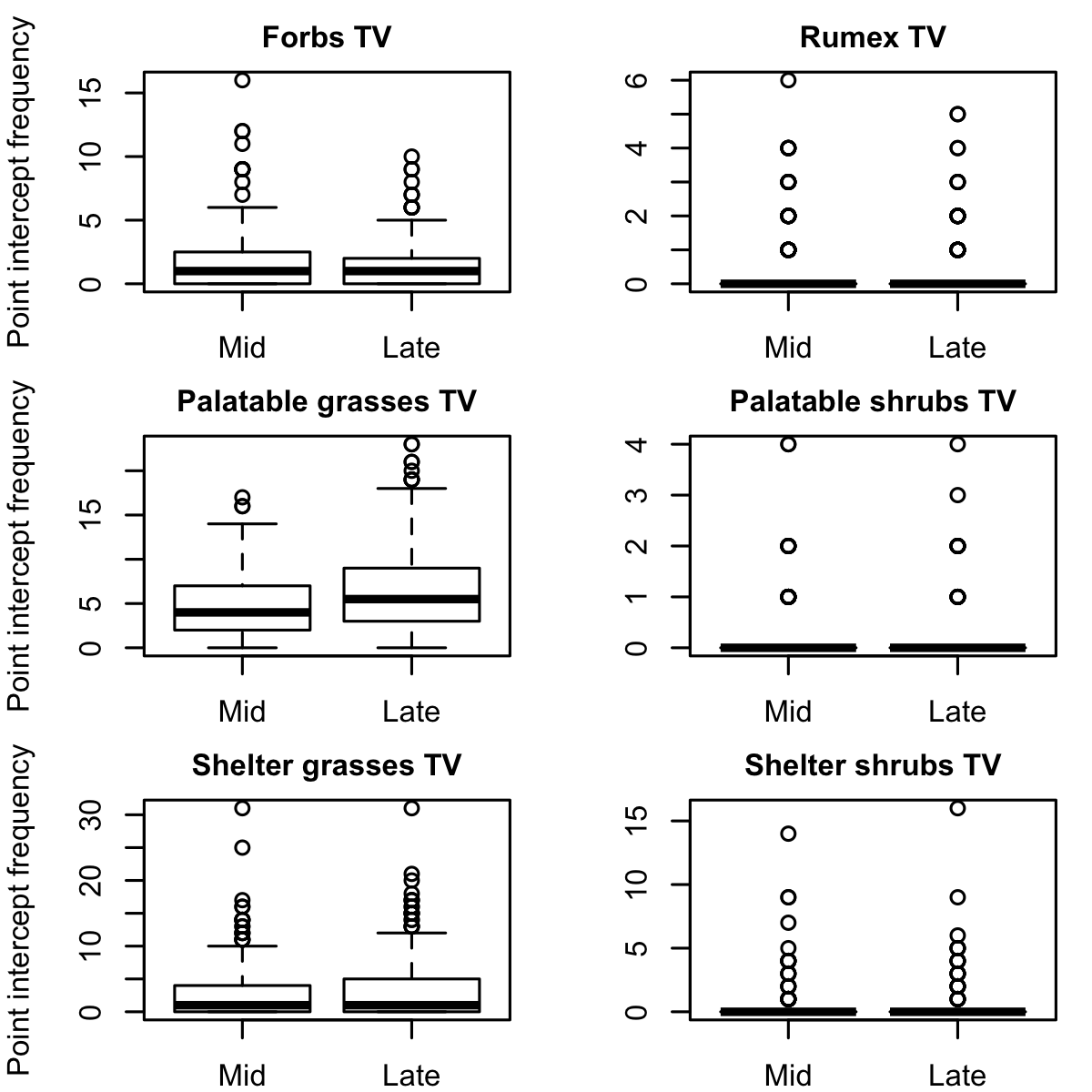
**

**Figure S4d**

**
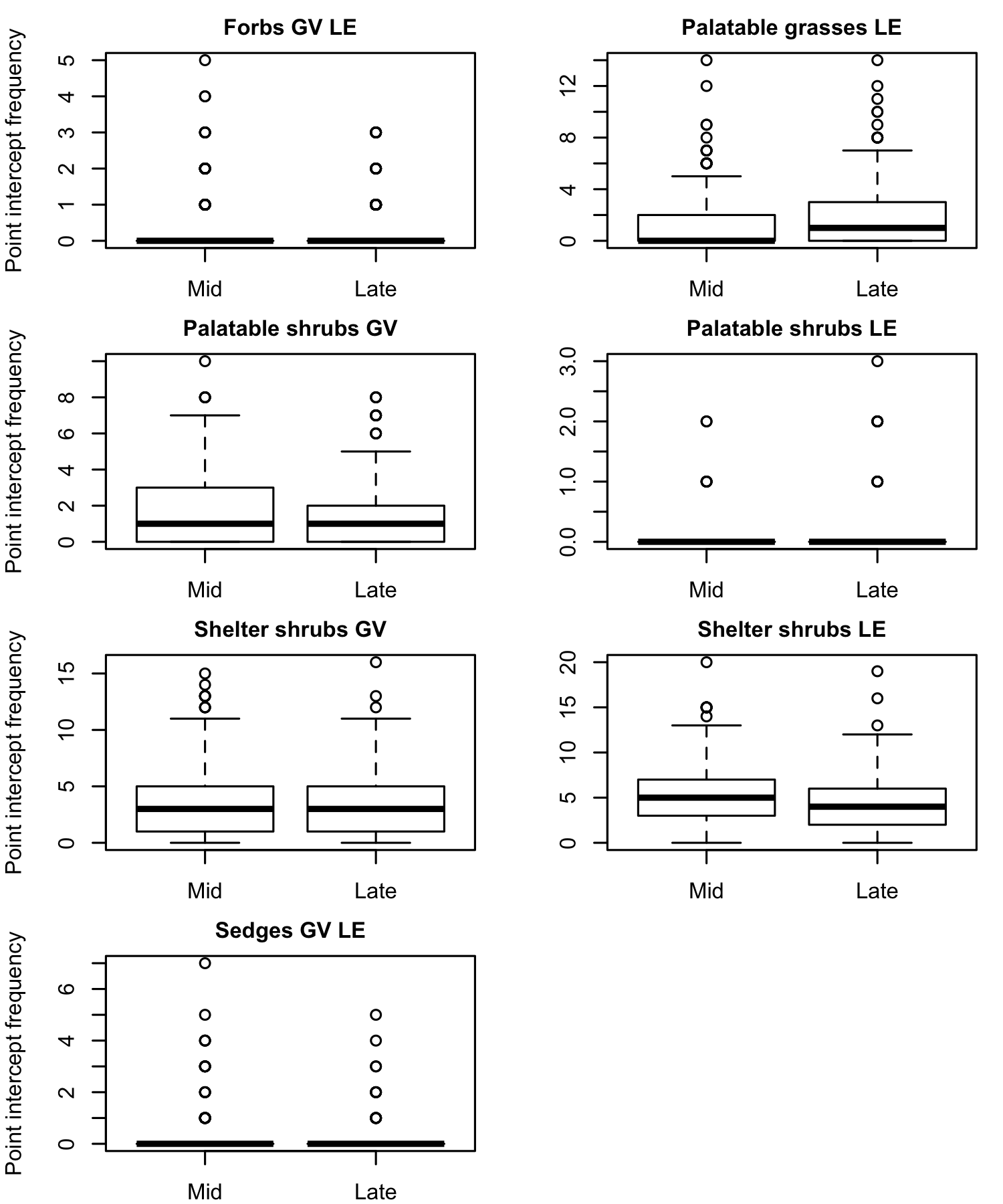
**

**Supplementary text S2: Analyses and results model averaging approach**

To assess whether model averaging would give similar results as the “best model” approach, we repeated the analyses for the trophic interaction models using a set of candidate models (presented in Supplementary material Appendix S4, Table S9). We used model averaging approach based on AIC to achieve parameter estimates for the predictor variables (function model.avg in R package MuMIN)(Bartoń 2013).

**Supplementary Table S3.** Model averaged parameter estimates for tundra vole, grey-sided vole and lemming abundance during the first peak. Column importance shows the relative importance of each variable (sum of AIC weights in which the model appears). Predictor variables which 95% confidence intervals do not cross zero are denoted with bold. Estimates are given at log-scale.

| Species |  | Estimate | 95% CI | Importance |
| --- | --- | --- | --- | --- |
| Tundra vole | Intercept | 1.19 | -0.41, 2.97 |  |
|  | **Forbs** | **-0.53** | **-0.98, -0.07** | **1.00** |
|  | **Palatable grasses** | **0.46** | **0.14, 0.79** | **1.00** |
|  | **Shelter grasses** | **0.17** | **0.06, 0.29** | **1.00** |
|  | Shelter shrubs | -0.01 | -0.08, 0.06 | 0.07 |
|  | Palatable shrubs | 0.01 | -0.06, 0.07 | 0.07 |
|  | Rumex | 0.00 | -0.03, 0.03 | 0.01 |
| Grey-sided vole | Intercept | -1.57 | -4.53, 1.38 |  |
|  | **Shelter shrubs** | **0.57** | **0.02, 1.11** | **0.96** |
|  | Forbs | -0.03 | -0.22, 0.16 | 0.26 |
|  | Palatable shrubs | 0.00 | -0.10, 0.11 | 0.04 |
| Lemming | Intercept | 1.44 | 0.44, 2.44 |  |
|  | **Palatable shrubs** | **0.32** | **0.09, 0.54** | **1.00** |
|  | **Palatable grasses** | **-0.46** | **-0.69, -0.23** | **0.99** |
|  | Sedges | 0.03 | -0.18, 0.24 | 0.27 |
|  | Forbs | 0.00 | -0.12, 0.11 | 0.06 |
|  | Shelter shrubs | -0.01 | -0.18, 0.16 | 0.02 |

**Supplementary text S3: Model selection second peak**

To assess the relationship between plant functional group biomass and rodent abundance during the second peak, we run model selection for the dataset of the second peak. For tundra voles and lemmings, null models were unanimously ranked as the best model (Table S4). For grey-sided voles, also two other models received substantial support (Table S4).

**Supplementary Table S4.** Model selection table for second peak for tundra voles (based on AICc, i.e. corrected Akaike Information criterion), grey-sided voles (based on QAICc, i.e. . corrected Akaike Information criterion for overdispersed data, ĉ =~ 1.83) and lemmings (based on AICc). K = number of parameters in the model, δAICc = difference between the present model and the best model, AICcWt = Akaike weight for AICc. Models within δAICc<2 (i.e. no strong clear difference in support for the different models (Burnham and Anderson 2004)) are denoted in bold.

| Species | Predictor variables | K | AICc | δAICc | AICcWt |
| --- | --- | --- | --- | --- | --- |
| Tundra vole | **Null model** | **1** | **157.29** | **0.00** | **0.57** |
|  | **Forbs+ shelter shrubs + palatable shrubs** | **4** | **158.76** | **1.47** | **0.27** |
|  | forbs + palatable grasses + shelter grasses | 4 | 160.40 | 3.10 | 0.12 |
|  | Forbs+ shelter shrubs + palatable shrubs + palatable grasses + shelter grasses | 6 | 163.32 | 6.02 | 0.03 |
|  | Rumex+ Forbs+ shelter shrubs + palatable shrubs + palatable grasses + shelter grasses | 7 | 166.54 | 9.25 | 0.01 |
| Grey-sided vole | **Shelter shrubs** | **3** | **74.60** | **0.00** | **0.39** |
|  | **Forbs + Shelter shrubs** | **4** | **75.46** | **0.86** | **0.25** |
|  | **Null model** | **2** | **75.97** | **1.37** | **0.20** |
|  | Palatable shrubs | 3 | 77.67 | 3.07 | 0.08 |
|  | Forbs + palatable shrubs | 4 | 77.88 | 3.28 | 0.08 |
| Lemming | **Null model** | **1** | **119.31** | **0.00** | **0.55** |
|  | Palatable grasses + palatable shrubs + sedges | 4 | 121.83 | 2.52 | 0.16 |
|  | Palatable shrubs + shelter shrubs | 3 | 122.13 | 2.82 | 0.13 |
|  | Palatable shrubs + shelter shrubs + sedges + forbs | 5 | 123.79 | 4.48 | 0.06 |
|  | Palatable grasses + palatable shrubs | 3 | 123.98 | 4.67 | 0.05 |
|  | Palatable grasses + palatable shrubs + sedges + forbs | 5 | 124.81 | 5.50 | 0.03 |
|  | Palatable grasses + palatable shrubs + shelter shrubs + sedges + forbs | 6 | 126.48 | 7.17 | 0.02 |

**Supplementary Table S5.** Comparison of temporal transferability with all available quadrats and only quadrats included in the dataset during the first peak. Column “Error” refers to type of predictive error (mean absolute prediction error, unit is number of rodent individuals, see methods for exact definitions). “Raw” refers to prediction error_RAW_ (i.e. using raw abundances) and “Relative” to prediction error_RELATIVE_ (i.e. error for relative differences in rodent numbers between sampling quadrates irrespective of potential differences in the average yearly density). Column “All quadrats” refers to mean ± standard deviation of predictive error based on “best” model for each species (cf. Table 3) including all available quadrates (i.e. corresponding to values in Table 4). Column “Only quadrats from 1^st^ peak” gives same statistics for a dataset covering only sampling quadrats that were sampled during both peaks.

| Species | Error | All quadrats (n =28) | Only quadrats from 1^st^ peak (n = 19) |
| --- | --- | --- | --- |
| Tundra vole | Raw | 5.26 ±5.52 | 4.87 ±6.46 |
|  | Relative | 4.19 ±5.72 | 4.44 ±6.65 |
| Grey-sided vole | Raw | 3.13 ±2.02 | 2.87 ±2.2 |
|  | Relative | 2.38 ±1.70 | 2.4 ±1.74 |
| Lemming | Raw | 2.16 ±1.58 | 2.26 ±1.83 |
|  | Relative | 2.18 ±1.53 | 2.32 ±1.64 |

**Appendix S3: Additional information on plant functional group biomass data**

**Supplementary text S4: Rodent species-specific plant functional groups**

For all rodent species, we included most important food items based on studies of the focal populations’ diet (Soininen et al. 2013b, Soininen et al. 2013c) and plant groups that are able to provide shelter.

For tundra voles, we included forbs (the most preferred food item group), but separated *Rumex* *acetosa* coll. from other forbs, as this species alone composes on average 38% of tundra vole diets in the focal population. Tundra voles prefer grasses as a group, but feed very little on silica rich grasses (Appendix S3; Supplementary text S5, Soininen 2012). We therefore divided grasses into two groups; silica-poor grasses that provide mainly food as they do not form tussocks, and tussock-forming silica-rich grasses that provide mainly shelter. Within shrubs, tundra voles prefer willows (Salicaceae), but not other shrubs. We therefore separated also shrubs into those that provide food and shelter (Salicaceae) and those that provide mainly shelter (all others shrubs).

For grey-sided voles, we included forbs (the most preferred food item group). Grey-sided voles select for ericoid shrubs, of which deciduous species are most commonly eaten. We therefore divided shrubs into those that provide food and shelter (deciduous ericoids) and those that provide mainly shelter (all others shrubs). Grasses compose a small part of the average grey-sided vole diets (4%) and selectivity for grasses varies from positive to negative between individuals of the species. As the biomass of tussock-forming grasses is very low in the focal heath quadrates, we excluded grasses from the analyses for grey-sided voles.

For lemmings, we included forbs, as they were relatively frequent in lemming diets (mean proportion in diets = 0.11 (95% quantiles 0.00, 0.88)). Grasses (Poaceae) composed on average more than half of the diets (mean proportion in diets = 0.51 (95% quantiles 0.01, 0.99)). However, silica-rich species were very rarely recorded in the stomachs of the analysed individuals, and we therefore included only silica-poor in the analyses. Similarly to the grey-sided voles, we excluded shelter grasses from the analyses as biomass of tussock-forming grasses is very low in the focal heath sampling quadrats. Further, we included sedges and allies (Cyperaceae and Juncaceae) as a potential food plant group (mean proportion in diets = 0.13 (95% quantiles 0.00, 0.85). Among shrubs, willows (Salicaceae) were the only group included in the diets on average more than 10% (mean proportion in diets = 0.10 (95% quantiles 0.00, 0.88)). We therefore divided shrubs into palatable shrubs composed of Salicaceae, and shelter shrubs composed of Ericaceae (mean proportion in diets = 0.06 (95% quantiles 0.00, 0.49)) and Betulaceae (mean proportion in diets = 0.05 (95% quantiles 0.00, 0.51)). However, we were not able to include mosses, an important food plant group for the Norwegian lemming (Soininen et al. 2013c), in our analyses as we have no data on moss biomass in the sampling quadrats.

**Supplementary text S5: Analyses of tundra vole food preference within Poaceae**

We analysed tundra vole selectivity for different grass genera, using material published by Soininen et al. (2013b). The material consists in total of 66 tundra voles, which stomach contents were analysed using DNA metabarcoding of the chloroplast trnL barcode (with *g-h* primer pair, (Taberlet et al. 1991, Taberlet et al. 2007)). All individuals were collected by snap-trapping in 2007 from the sampling quadrates of study areas VJ and KO. We here included only the individuals for which we could assign at least 90% of their total Poaceae consumption to genera (n=46 out of 66). To determine selectivity, we compared grass genera-specific proportions within a vole individuals’ diet to the corresponding proportions from the sampling quadrat where the vole was trapped. We used compositional analysis of centered log-ratio transformed proportions as described by Soininen et al. (2013b). To test whether selectivity for different grass genera was significantly different, we computed pairwise significances in preference among food items using Wilks lambda, as described by Soininen et al. (2013b). Results of these significance tests are presented in Table S5.

**Supplementary Table S6**: Tundra vole (n=46) selectivity within Poaceae at plant genera level. A "+" sign indicates that the grass genus presented in the row was selected more than the corresponding genus presented in the column, "-" that it was less selected. Tripled sign indicates significant differences. Numbers in parentheses refer to mean silica (SiO_2_) concentration (proportion of dry weight) analysed by Soininen et al. (2013a) based on samples collected from herbivore exclosures in the same sampling quadrats where small rodent abundance and plant biomass was analysed for. Analyses were done for the most common species present in the study area for each genus. Values marked with * are for genera for which data on silica content are based on Smis et al. (2014) data from Northern Norway, while ** indicates data from (Hodson et al. 2005, global review, data for species present in study area only).

|  | Agr  (2.89)* | Ant (1.42) | Ave (1.05) | Cal (2.99) | Des (2.33) | Fes (1.56)* | Nar (4.09) | Phl (0.63) | Poa (3.29)** |
| --- | --- | --- | --- | --- | --- | --- | --- | --- | --- |
| Agr | 0 | + | --- | --- | + | - | +++ | ---- | --- |
| Ant | - | 0 | --- | --- | + | --- | +++ | --- | --- |
| Ave | +++ | +++ | 0 | - | +++ | + | +++ | + | - |
| Cal | +++ | +++ | + | 0 | +++ | + | +++ | + | + |
| Des | - | - | --- | --- | 0 | --- | +++ | --- | --- |
| Fes | + | +++ | - | - | +++ | 0 | + | + | - |
| Nar | --- | --- | --- | --- | - | --- | 0 | --- | --- |
| Phl | +++ | +++ | - | - | +++ | - | +++ | 0 | - |
| Poa | +++ | +++ | + | - | +++ | + | +++ | + | 0 |

Agr= *Agrostis*, Ant = *Antoxanthum*, Ave = *Avenella*, Cal = *Calamagrostis*, Des = *Deschampsia*, Fes = *Festuca*, Nar = *Nardus*, Phl = *Phleum*, Poa = *Poa*.

Among grasses, silica-rich genera, i.e. *Deschampsia* and *Nardus*, were the least preferred by tundra voles. Within these genera, only one species is present (*D.cespitosa* and *N. stricta*, respectively) in the study area. On the other hand, *Calamagrostis* was the most preferred genera. *Calamagrostis phragmitoides* is equally silica-rich as *D*. *cespitosa* (Soininen et al. 2013a). While the silica content of other *Calamagrostis* species present in the study area (*C. lapponum* and *C. neglecta*) is unknown, silica content of *Calamagrostis* range from high to intermediate (Hodson et al. 2005, Smis et al. 2014). No *Calamagrostis* in the tundra vole diet data had species level resolution.

**Supplementary Table S7.** Plant biomass in meadow habitat and plant functional group composition for analyses of tundra vole abundance. Included are data used for the first peak; for each quadrat either year 2006 or 2007, depending which year the number of voles was higher. Species with lower mean biomass than 0.01g/m^2^ are excluded from the table. Forbs marked with * were categorised as large forbs, others as small forbs. When two species within a genera could not always be distinguished with confidence, data is combined and the species names separated with /. Within thicket forming *Salix* species were pooled to “Salix green-leaved” (*S. phylicipholia*), “*Salix* grey-leaved” (*S. lanata*) and “*Salix* grey-green-leaved” (*S. glauca, S.hastata, S.lapponum* and all potential hybrids of *Salix*, unless they were recorded as *Salix* sp. in the field). Group “Excluded” consists of plant taxa that were not included in any of the functional groups used in the analyses (see Supplementary text S4 for details).

| Functional group | Plant species | Mean (±SD) |
| --- | --- | --- |
| Forbs_TV_ | *Alchemilla alpina* | 0.07 (0.29) |
|  | *Alchemilla* sp. | 3.93 (4.58) |
|  | *Angelica archangelica** | 0.30 (1.14) |
|  | *Anthriscus sylvestris** | 0.03 (0.15) |
|  | *Bistorta vivipara* | 3.22 (2.06) |
|  | *Botrychium lunaria* | 0.03 (0.17) |
|  | *Caltha palustris* | 0.08 (0.30) |
|  | *Campanula rotundifolia* | 0.01 (0.04) |
|  | *Cerastium cerastoides* | 0.01 (0.04) |
|  | *Cerastium fontanum/alpinum* | 0.11 (0.19) |
|  | *Chamerion angustifolium** | 0.21 (0.53) |
|  | *Chamaepericlumenum suececicum* | 1.23 (4.51) |
|  | *Cirsium heterophyllum** | 1.51 (2.91) |
|  | *Comarum palustre* | 0.51 (1.21) |
|  | *Epilobium hornemannii* | 0.20 (0.49) |
|  | *Epilobium palustre* | 0.03 (0.11) |
|  | *Epilobium* sp. | 0.15 (0.45) |
|  | *Geranium sylvaticum** | 1.66 (3.45) |
|  | *Hieracium* sect *Alpina* | 0.02(0.10) |
|  | *Equisetum arvense/pratense* | 2.91 (2.53) |
|  | *Equisetum pratense* | 0.01 (0.04) |
|  | *Equisetum* sp. | 0.04 (0.08) |
|  | *Equisetum sylvaticum* | 3.30 (9.14) |
|  | *Equisetum variegatum* | 0.01 (0.04) |
|  | *Euphrasia wettsteinii* | 0.09 (0.24) |
|  | *Myosotis* sp. | 0.01 (0.04) |
|  | *Omalotheca norvegia* | 0.21 (0.33) |
|  | *Omalotheca supina* | 0.04 (0.15) |
|  | *Parnassia palustris* | 0.01 (0.06) |
|  | *Pyrola minor* | 0.40 (0.57) |
|  | *Ranunculus acris* | 1.35 (1.33) |
|  | *Rhinantus minor* | 0.31( 0.68) |
|  | *Rhodiola rosea* | 0.07 (0.23) |
|  | *Rubus chamaemorus* | 0.21 (0.75) |
|  | *Sagina saginoides* | 0.02 (0.07) |
|  | *Saussurea alpine* | 0.03 (0.17) |
|  | *Sibbaldia procumbens* | 0.14 (0.33) |
|  | *Solidago virgaurea* | 1.23 (1.78) |
|  | *Stellaria borealis* | 0.02 (0.09) |
|  | *Stellaria media* | 0.01 (0.02) |
|  | *Stellaria nemorum* | 0.45 (0.59) |
|  | *Stellaria* sp. | 0.02 (0.04) |
|  | *Taraxacum sp* | 0.51 (0.77) |
|  | *Trientalis europaeae* | 0.61 (0.47) |
|  | *Trientalis* sp. | 0.06 (0.26) |
|  | *Trollius europaeus** | 2.45 (3.69) |
|  | *Veronica alpine* | 0.01 (0.02) |
|  | *Veronica* sp. | 0.01 (0.04) |
|  | *Viola* sp. | 3.95 (3.14) |
| Rumex | *Rumex acetosa* | 8.43 (6.41) |
| Palatable grasses_TV_ | *Antoxanthum nipponicum* | 11.15 (15.86) |
|  | *Agrostis* sp. | 0.39 (1.18) |
|  | *Avenella flexuosa* | 21.15 (19.50) |
|  | *Calamagrostis neglecta/lapponica* | 9.18 (11.23) |
|  | *Festuca* sp. | 2.63 (3.68) |
|  | *Milium effusum* | 0.34 (1.62) |
|  | *Phleum alpinum* | 1.40 (1.39) |
|  | *Poa alpina* | 0.12 (0.31) |
|  | *Poa* sp. | 4.67 (4.18) |
|  | *Vahlodea atropururea* | 0.15 (0.50) |
| Palatable shrubs_TV_ | *Salix* green-leaved | 13.93 (24.81) |
|  | *Salix* grey-leaved | 11.80 (21.51) |
|  | *Salix* grey-green-leaved | 10.72 (17.31) |
|  | *Salix herbaceae* | 3.85 (5.89) |
|  | *Salix* sp. | 7.63 (11.12) |
| Shelter shrubs_TV_ | *Arctostaphylos alpina* | 0.02 (0.11) |
|  | *Betula nana* | 9.11 (18.45) |
|  | *Empetrum nigrum* | 5.64 (12.64) |
|  | *Vaccinium myrtillus* | 2.15 (4.66) |
|  | *Vaccinium uliginosum* | 1.65 (3.07) |
|  | *Vaccinium vitis-idaea* | 1.78 (4.34) |
| Shelter grasses_TV_ | *Deschampsia cespitosa* | 21.50 (24.77) |
| Excluded | *Calamagrostis phragmitoides* | 10.52 (11.73) |
|  | *Carex aquatilis* coll. | 1.31 (5.19) |
|  | *Carex bigelowii* | 0.52 (1.54) |
|  | *Carex brunnescens/canescens* | 1.70 (2.68) |
|  | *Carex lachenalii* | 0.17 (0.51) |
|  | *Carex nigra* coll. | 4.40 (8.51) |
|  | *Carex rarifolia* | 0.01 (0.04) |
|  | *Carex* sp. | 1.41 (1.75) |
|  | *Carex vaginata* | 0.16 (0.62) |
|  | *Eriophorium angustifolium* | 0.03 (0.14) |
|  | *Eriophorium* sp. | 0.07 (0.36) |
|  | *Juniperus communis* | 0.15 (0.73) |
|  | *Juncus filiformis* | 0.51 (1.22) |
|  | *Luzula multiflora* | 0.19 (0.31) |
|  | *Luzula sudetica* | 0.08 (0.26) |
|  | *Lycopodium clavatum* coll. | 0.01 (0.04) |
|  | *Nardus stricta* | 0.66 (2.01) |
|  | *Selaginella selaginoides* | 0.05 (0.08) |

**Supplementary Table S8.** Plant biomass in heath habitat and plant functional group composition for analyses of grey-sided vole and lemming abundance. Included are data used for the first peak for each rodent species separately; either year 2006 or 2007, depending which year the species peaked in a given quadrat. Species with lower mean biomass than 0.01g/m^2^ are excluded from the table. When two species within a genera could not always be distinguished with confidence, data is combined and the species names separated with /. Within thicket forming *Salix* species were pooled to “Salix green-leaved” (*S. phylicipholia*), “*Salix* grey-leaved” (*S. lanata*) and “*Salix* grey-green-leaved” (*S. glauca, S.hastata, S.lapponum* and all potential hybrids of *Salix*, unless they were recorded as *Salix* sp. in the field). Group “Excluded” consists of plant taxa that were not included in any of the functional groups used in the analyses (see Supplementary text S4 for details).

| Functional group  grey-sided voles | Functional group  lemmings | | | Plant species | | | | Mean (±SD)  voles | Mean (±SD)  lemming |
| --- | --- | --- | --- | --- | --- | --- | --- | --- | --- |
| Forbs_GV_ | Forbs_LE_ | | | *Alchemilla* sp. | | | | 0.03 (0.15) | 0.03 (0.42) |
|  |  | | | *Alchemilla alpine* | | | | - | 0.08 (0.42) |
|  |  | | | *Bartsia alpina* | | | | 0.01 (0.04) | - |
|  |  | | | *Bistorta vivipara* | | | | 0.23 (0.80) | 0.24 (0.81) |
|  |  | | | *Cerastium* sp. | | | | 0.01 (0.04) | 0.01 (0.04) |
|  |  | | | *Chamaepericlumenum suececicum* | | | | 4.54 (4.82) | 4.48 (4.81) |
|  |  | | | *Cirsium heterophyllum* | | | | 0.03 (0.14) | 0.03 (0.14) |
|  |  | | | *Equisetum arvense/pratense* | | | | 0.08 (0.37) | - |
|  |  | | | *Equisetum sylvestris* | | | | 0.06 (0.22) | 0.05 (0.18) |
|  |  | | | *Erigeron angustifolium* | | | | 0.09 (0.47) | 0.14 (0.50) |
|  |  | | | *Geranium sylvaticum* | | | | 0.08 (0.26) | 0.08 (0.26) |
|  |  | | | *Hierachium sect Alpina* | | | | 0.04 (0.14) | 0.05 (0.14) |
|  |  | | | *Hierachium* sp. | | | | 0.01 (0.04) | - |
|  |  | | | *Linnea borealis* | | | | 0.02 (0.05) | 0.01 (0.07) |
|  |  | | |  | | | |  |  |
|  |  | | | *Omalotheca norvegia* | | | | 0.01 (0.04) | 0.01 (0.04) |
|  |  | | | *Pyrola minor* | | | | 0.04 (0.13) | 0.01 (0.04) |
|  |  | | | *Ranunculus acris* coll. | | | | 0.10 (0.36) | 0.10 (0.36) |
|  |  | | | *Rhinantus minor* coll. | | | | 0.03 (0.13) | 0.02 (0.12) |
|  |  | | | *Rubus chamaemorus* | | | | 0.24 (0.78) | 0.20 (0.74) |
|  |  | | | *Rumex acetosa* coll. | | | | 0.01 (0.05) | 0.01 (0.05) |
|  |  | | | *Solidago virgaurea* | | | | 0.25 (0.46) | 0.32 (0.70) |
|  |  | | | *Taraxacum* sp. | | | | 0.01 (0.04) | 0.01 (0.04) |
|  |  | | | *Trientalis europaeae* | | | | 0.14 (0.23) | 0.16 (0.24) |
|  |  | | | *Trollius europaeus* | | | | 0.04 (0.19) | - |
|  |  | | | *Viola* sp. | | | | 0.03 (0.15) | 0.03 (0.15) |
| Palatable shrubs_GV_ | Shelter shrubs_L_ | | | *Vaccinium myrtillus* | | | | 68.18 (32.01) | 69.18 (36.29) |
|  |  | | | *Vaccinium uliginosum* | | | | 10.57 (9.67) | 10.27 (9.88) |
|  |  | | | *Arctostaphylos alpina* | | | | 2.76 (6.28) | 3.00 (6.52) |
|  |  | | | *Loiseleuria procumbens* | | | | 2.01 (5.93) | 2.06 (5.92) |
| Shelter shrubs_GV_ |  | | | *Betula nana* | | | | 215.89 (109.87) | 217.69 (106.46) |
|  |  | | | *Empetrum nigrum*  *hermaphroditum* | | | | 164.71 (105.03) | 174.63 (112.94) |
|  |  | | | *Vaccinium vitis-idaea* | | | | 19.45 (13.32) | 18.58 (11.97) |
|  |  | | | *Betula pubescens* coll. | | | | 3.89 (13.48) | 2.04 (10.18) |
|  |  | | | *Phyllodoce caerulea* | | | | 0.86 (2.41) | 0.67 (1.64) |
|  |  | | | *Andromeda polifolia* | | | | 0.49 (2.22) | 0.44 (2.21) |
|  |  | | | *Harimanella hypnoides* | | | | 0.01 (0.02) | - |
|  | Palatable shrubs_L_ | | | *Salix* green-leaved | | | | 0.14 (0.68) | 0.14 (0.68) |
|  |  | | | *Salix* grey-green-leaved | | | | 0.05 (0.23) | 0.04 (0.23) |
|  |  | | | *Salix herbaceae* | | | | 4.30 (7.24) | 4.56 (7.55) |
|  |  | | | *Salix* sp. | | | | 0.10 (0.45) | 0.09 (0.45) |
| Excluded | Palatable grasses_L_ | | | *Avenella flexuosa* | | | | 10.18 (8.49) | 10.36 (8.99) |
|  |  | | | *Festuca* sp. | | | | 0.38 (0.88) | 0.54 (1.08) |
|  |  | | | *Antoxanthum nipponicum* | | | | 0.25 (1.10) | 0.24 (1.10) |
|  |  | | | *Agrostis* sp. | | | | 0.03 (0.18) | 0.04 (0.18) |
|  |  | | | *Phleum alpinum* | | | | 0.01 (0.03) | 0.01 (0.03) |
|  |  | | | *Trisetum spicatum* | | | | - | 0.01 (0.03) |
|  | Sedges | | | *Carex aquatilis* coll. | | | | 0.02 (0.08) | 0.08 (0.39) |
|  |  | | | *Carex bigelowii* | | | | 0.81 (2.49) | 0.59 (2.06) |
|  |  | | | *Carex limosa x rariflora* | | | | 0.19 (0.97) | 0.19 (0.96) |
|  |  | | | *Carex rariflora* | | | | 0.19 (0.97) | 0.19 (0.96) |
|  |  | | | *Carex* sp. | | | | 0.28 (1.04) | 0.32 (1.08) |
|  |  | | | *Carex vaginata* | | | | 0.05 (0.25) | 0.04 (0.22) |
|  |  | | | *Juncus filiformis* | | | | 0.04 (0.21) | - |
|  |  | | | *Juncus trifidus* | | | | 0.62 (1.03) | 0.63 (0.96) |
|  |  | | | *Luzula multiflora* | | | | 0.01 (0.04) | 0.01 (0.04) |
|  |  | | | *Luzula* sp. | | | | 0.03 (0.12) | 0.03 (0.12) |
|  |  | | | *Luzula sudetica* | | | | 0.01 (0.04) | 0.01 (0.04) |
|  | Excluded | | | *Calamagrostis phragmitoides* | | | | 0.07 (0.26) | 0.07 (0.26) |
|  |  | | | *Deschamspia cespitosa* | | | | 0.18 (0.82) | 0.18 (0.82) |
|  |  | | | *Nardus stricta* | | | | 0.63 (1.48) | 0.63 (1.48) |
|  |  | | | *Lycopodium annotinum* | | | | 0.01 (0.04) | - |
|  | |  | | | |  |  |  |  |
|  | | |  | |  | |  |  |  |

**Appendix S4. Additional details on methodology**

**Supplementary text S6: Additional details on snap-trapping methodology**

Rodent abundance in the sampling quadrats was assessed using the method described by Myllymäki et al. (1971). We used three snap traps that were selectively set (i.e. on vole runways or in from of tunnels) at radius of 2 meters in each of the four corners of the quadrat. The traps were baited with raisins (*Vitis vinifera*) and oat flakes (*Avena sativa*). The traps were open over two consecutive nights. The traps were visited daily, trapped rodents collected, and bait renewed if necessary. This resulted as 24 trap-nights per quadrat per trapping session (i.e. July or September). We used the number of trapped rodents per species per quadrat per trapping session as an index for local population abundance.

**Supplementary text S7: Estimation of density dependence**

Intraspecific density dependence for the three species was estimated based on a state-space model where measurement error was explicitly incorporated by using the two trapping days (j) for each sampling quadrat (i) within a trapping season (k=1:2) as temporal removal occasions (Kéry and Royle 2016), assuming the population to be closed over the two days. The models were run through software R, using the JAGS package rjags (Plummer 2016).

In the model, we assumed the observed trapping data ($y_{i,j}$) to come from a poisson distribution with poisson parameter *pi*:

$$y_{i,j}\sim poisson({pi}_{i,j})$$

Hence, we regarded each element of the multinomial trapping data vector as an independent poisson count with expected value λ_i_π_j_, where λ = expected abundance, π = multinomial cell probability, and p = detection probability:

$$\pi_{i,1}=p_{i}*\lambda_{i}$$

$${\pi i}_{i,2}=p_{i}(1-p_{i})*\lambda_{i}$$

We modelled the expected abundance in autumn $(\lambda_{i,k=2})$ using a log-linear model with an intercept ($\beta_{0}$) and interspecific density dependence ($\beta_{DD}$), based on the expected abundance from the spring ($\lambda_{i,k=1}$):

$$\log\left( \lambda_{i,k=2} \right)=\beta_{0}+\beta_{DD}* \lambda_{i,k=1}$$

We modelled detection probability (p) using a logit-linear model with an intercept allowed to wary across sampling quadrats (α_i_):

$$p_{i}=\alpha_{i}$$

Note that the expected abundance for the spring data (k=1) was modelled in the same way, except that the expected abundance was modelled with just an intercept allowed to vary across sampling quadrats ($\beta_{0,i}$).

**Supplementary text S8: Simulation of minimum obtainable prediction error**

Because of the poisson distributed response variable, difference between observed and predicted values even for 100% predictive power would never reach zero (Cox and Wermuth 1992). Thus, the evaluation of model transferability needs to be related to what is the minimum obtainable error for a perfectly transferable model. In order to evaluate the measures of predictive ability obtained for our models, we used simulations to determine the minimum obtainable prediction error, given the range of abundance and sample sizes in the current study. We did this by using random generations from the poisson distribution (function *rpois* in R) with parameter lambda,where lambda was a sequence of abundances from the minimum to the maximum in our data. We extracted the predicted values from a *glm* of the random generations (response) against the sequence of abundances from the minimum to the maximum in our data (predictor). We then calculated the two measures of mean absolute prediction error (prediction error_RAW_ and prediction error _RELATIVE_) as described in the main text.

**Supplementary Table S9.** Model selection table first peak for tundra voles, lemmings (based on AICc, i.e. corrected Akaike Information criterion), and grey-sided voles (based on QAICc, i.e. corrected Akaike Information criterion for overdispersed data, ĉ =~ 1.54). K = number of parameters in the model, δAICc = difference between the present model and the best model, AICcWt = Akaike weight for AICc. The best models, i.e. models with lowest AICc/ QAICc, are denoted in bold.

| Species | Predictor variables | K | AICc | δAICc | AICcWt |  |
| --- | --- | --- | --- | --- | --- | --- |
| Tundra vole | **forbs + palatable grasses + shelter grasses** | **4** | **117.26** | **0.00** | **0.93** |  |
|  | forbs+ shelter shrubs + palatable shrubs + palatable grasses + shelter grasses | 6 | 122.78 | 5.52 | 0.06 |  |
|  | *Rumex*+ forbs+ shelter shrubs + palatable shrubs + palatable grasses + shelter grasses | 7 | 126.70 | 9.44 | 0.01 |  |
|  | Null model | 1 | 137.13 | 19.87 | 0.00 |  |
|  | forbs+ shelter shrubs + palatable shrubs | 4 | 138.66 | 21.40 | 0.00 |  |
| Grey-sided vole | **shelter shrubs** | **3** | **79.42** | **0.00** | **0.65** |  |
|  | forbs + shelter shrubs | 4 | 81.61 | 2.19 | 0.22 |  |
|  | Null model | 2 | 83.43 | 4.01 | 0.09 |  |
|  | palatable shrubs | 3 | 86.01 | 6.59 | 0.02 |  |
|  | forbs + palatable shrubs | 4 | 86.92 | 7.49 | 0.02 |  |
| Lemming | **palatable grasses + palatable shrubs** | **3** | **96.60** | **0.00** | **0.73** |  |
|  | palatable grasses + palatable shrubs + sedges | 4 | 99.13 | 2.54 | 0.21 |  |
|  | palatable grasses + palatable shrubs + sedges + forbs | 5 | 102.29 | 5.69 | 0.04 |  |
|  | palatable grasses + palatable shrubs + shelter shrubs + sedges + forbs | 6 | 104.35 | 7.76 | 0.02 |  |
|  | palatable shrubs + shelter shrubs + sedges + forbs | 5 | 106.13 | 9.54 | 0.01 |  |
|  | palatable shrubs + shelter shrubs | 3 | 113.84 | 17.24 | 0.00 |  |
|  | Null model | 1 | 119.53 | 22.93 | 0.00 |  |

**References Supplementary Material Appendix S1-S4**

Bartoń, K. 2013. MuMIn: multi-model inference* R package 1.9.5.

Burnham, K. P., and D. R. Anderson. 2004. Multimodel inference - understanding AIC and BIC in model selection. Sociological Methods & Research **33**:261-304.

Cox, D. R., and N. Wermuth. 1992. A comment on the coefficient of determination for binary responses. American Statistician **46**:1-4.

Hodson, M. J., P. J. White, A. Mead, and M. R. Broadley. 2005. Phylogenetic Variation in the Silicon Composition of Plants. Annals of Botany **96**:1027-1046.

Kéry, M., and A. J. Royle. 2016. Applied hierarchical modeling in ecology: analysis of distribution, abundance and species richness in R and BUGS. 1 edition. Elsevier.

Myllymäki, A., A. Paasikalio, E. Pankakoski, and V. Kanevo. 1971. Removal experiments on small quadrats as a means of rapid assessment of the abundance of small mammals. Annales zoologici Fennici **8**:177-185.

Plummer, M. 2016. rjags: Bayesian Graphical Models using MCMC, version 4-6. <http://mcmc-jags.sourceforge.net>.

Smis, A., F. J. A. Murguzur, E. Struyf, E. M. Soininen, J. G. H. Jusdado, P. Meire, and K. A. Bråthen. 2014. Determination of plant silicon content with near infrared reflectance spectroscopy. Frontiers in Plant Science **5**.

Soininen, E. M. 2012. Interactions between small rodents and their food plants in tundra habitats (PhD thesis). University of Tromsø, Tromsø, Norway.

Soininen, E. M., K. A. Bråthen, J. G. H. Jusdado, S. Reidinger, and S. E. Hartley. 2013a. More than herbivory: induction of silica-based defences in grasses varies with plant species, genotype and location. Oikos **122**:30-41.

Soininen, E. M., V. T. Ravolainen, K. A. Bråthen, N. G. Yoccoz, L. Gielly, and R. A. Ims. 2013b. Arctic small rodents have diverse diets and flexible food selection PLoS ONE **8**:e68128.

Soininen, E. M., L. Zinger, L. Gielly, E. Bellemain, K. A. Bråthen, C. Brochmann, L. S. Epp, G. Gussarova, K. Hassel, J.-A. Henden, S. T. Killengreen, T. Rämä, H. K. Stenøien, N. G. Yoccoz, and R. A. Ims. 2013c. Shedding new light on the diet of Norwegian lemmings: DNA metabarcoding of stomach content. Polar Biology **36**:1069-1076.

Taberlet, P., E. Coissac, F. Pompanon, L. Gielly, C. Miquel, A. Valentini, T. Vermat, G. Corthier, C. Brochmann, and E. Willerslev. 2007. Power and limitations of the chloroplast *trn*L (UAA) intron for plant DNA barcoding. Nucleic Acids Research **35**:e14.

Taberlet, P., L. Gielly, G. Patou, and J. Bouvet. 1991. Universal primers for amplification of 3 noncoding regions of chloroplast DNA. Plant Molecular Biology **17**:1105-1109.
